# Tuning Germanane Band Gaps via Cyanoethyl Functionalization for Cutting-Edge Photoactive Cathodes: Photoenhanced Hybrid Zinc-Ion Capacitor Evaluation

**DOI:** 10.1021/acsami.3c17420

**Published:** 2024-03-18

**Authors:** Jalal Azadmanjiri, Jiri Sturala, Jakub Regner, Filipa M. Oliveira, Vlastimil Mazánek, Zdeněk Sofer

**Affiliations:** Department of Inorganic Chemistry, University of Chemistry and Technology Prague, Technická 5, 166 28 Prague 6, Czech Republic

**Keywords:** 2D materials, germanane, photoenhanced capacitors, photoactive cathodes, energy storage, Zn-ion capacitors

## Abstract

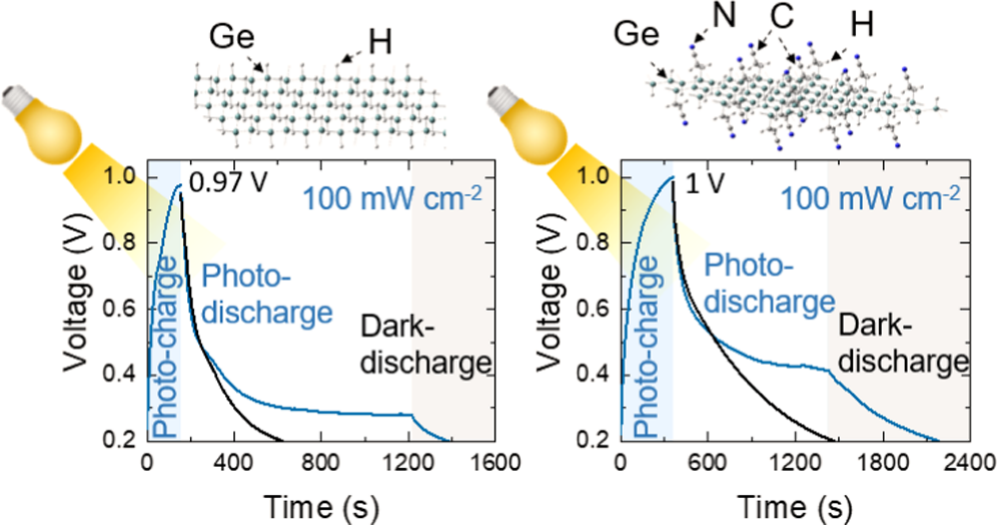

Energy harvesting and storing by dual-functional photoenhanced (photo-E) energy storage devices are being developed to battle the current energy hassles. In this research work, our investigations on the photoinduced efficiency of germanane (Ge–H) and its functionalized analogue cyanoethyl (Ge–C_2_–CN) are assessed as photocathodes in photo-E hybrid zinc-ion capacitors (ZICs). The evaluated self-powered photodetector devices made by these germanene-based samples revealed effective performances in photogenerated electrons and holes. The photo-E ZICs findings provided a photoinduced capacitance enhancement of ∼52% (for Ge–H) and ∼26% (for Ge–C_2_–CN) at a scan rate of 10 mV s^–1^ under 100 mW cm^–2^ illumination with 435 nm wavelength. Further characterizations demonstrated that the photo-E ZIC with Ge–C_2_–CN supply higher specific capacitance (∼6000 mF g^–1^), energy density (∼550 mWh kg^–1^), and power density (∼31,000 mW kg^–1^), compared to the Ge–H. In addition, capacitance retention of photo-E ZIC with Ge–C_2_–CN is ∼91% after 3000 cycles which is almost 6% greater than Ge–H. Interestingly, the photocharging voltage response in photo-E ZIC made by Ge–C_2_–CN is 1000 mV, while the photocharging voltage response with Ge–H is approximately 970 mV. The observed performances in Ge–H-based photoactive cathodes highlight the pivotal role of such two-dimensional materials to be applied as single architecture in new unconventional energy storage systems. They are particularly noteworthy when compared to the other advanced photo-E supercapacitors and could even be enhanced greatly with other suitable inorganic and organic functional precursors.

## 1 Introduction

The rapid fossil fuels depletion, environmental concerns, and the growing demand for clean energy sources have provoked research into next-generation energy storage technologies.^1−4^ As a green and renewable resource, solar energy possesses infinite prospects to be used in advanced energy applications.^5−7^ Therefore, extensive efforts are being carried out on different configurations of solar energy to generate innovative and effective energy conversion and/or storage devices.^5,8−10^ It is widely acknowledged that solar cells are capable of transforming solar energy into electricity.^11−14^ However, they face a limitation in terms of continuous energy storage. Thus, the integration of energy harvesting and energy-storing devices is one of the sensational forms of solar energy utilization.^15−18^ To address these attractive platforms and enhance their effective utilization in solar power, various solar photovoltaic systems have been integrated with other electrochemical energy storage solutions, including supercapacitors and batteries.^19−23^ Although integrated energy harvesting and storage devices exhibit impressive performance, they are surrounded by certain challenges. These include an energy mismatch between each section and essential supplementary exterior electronic devices, complex device architectures, a large Ohmic transport loss, substantial packaging space requirements, and elevated production expenses. To overcome some of these issues significant endeavors are being started to initiate how effective, low-dimensional, and more economical devices could be invented by new strategies and pioneer discovered materials.^24−26^ As a result, a dual-functional hypothesis structure has been introduced to use and enhance both energy harvesting and energy storage efficiency and reduce energy loss during transmission among individual modules.^27−29^ The operation of a dual-functional structure could be done by the generation of photoexcited electrons using solar energy absorption and then circulating the photogenerated electrons for storing energy.

In this context, materials with nanostructure dimensions offer unique advantages in terms of improved performance, efficiency, and versatility, making them crucial components in the development of more efficient and sustainable energy devices. Among different types of nanostructured materials, the family of two-dimensional (2D) materials demonstrates excellent physical, chemical, and mechanical properties that could be suitable for different types of advanced technologies.^30−33^ With the exclusion of compound 2D materials, their monoelementals (i.e., germanene, silicene, stanine, etc.)^34,35^ have garnered significant attention recently due to their exceptional electronic properties and the promise they hold for a wide range of applications.^36−38^

Germanane (Ge–H) with nonplanar sheets is a fully hydrogenated form of germanene with the formula of Ge_6_H_6_ and a sp^3^-hybridized 2D honeycomb-like lattice structure.^36,37^ It offers a high surface area and unique electronic characteristics. A distinguished feature of Ge–H is its tunable band gap which makes that material a prime candidate for applications requiring precise control over electronic properties.^39,40^ In the pursuit of optimizing Ge–H synthesis, its surface functionalization with other organic or inorganic precursors would be valuable to be examined and applied in diverse high-technology applications. Hence, in this research work, Ge–H and one of its functionalized analogues bearing the cyanoethyl (Ge–C_2_–CN) group are synthesized and then evaluated as photoactive cathode materials in photoenhanced hybrid zinc-ion capacitors (Photo-E ZICs). This will demonstrate an understanding of the fascinating characteristics of photoactive cathodes based on Ge–H and Ge–C_2_–CN materials and remark on how charge transfer occurs in these photoresponsive devices under both dark and illuminated conditions. In situations where the properties and mechanisms of these nanostructures are unclear or have not been explored in photoactive cathodes of photoenhanced energy storage devices, this research will shed light on them. It is observed that both samples are effective in facilitating efficient light absorption and energy conversion. In addition, carbon and nitrogen of C_2_–CN which are crucial elements in influencing the performance of energy storage devices are efficient in enhancing the electrical and electrochemical properties of electrodes.^41−43^ It was observed in this study that they improved capacitance, voltage response, energy and power densities, cycling life stability, and fast charge/discharge capabilities. This innovative approach takes advantage of the synergy between the inherent electronic properties of Ge–H and the specific characteristics imparted by the C_2_–CN groups. Employing the hybrid Zn-ion-based capacitors is due to the unique characteristics of Zn including high specific capacity (∼820 mAh g^–1^),^44^ low redox potential (–0.76 V vs standard hydrogen electrode),^45^ great stability,^46^ low cost,^47^ and safety.^48^ These features make ZICs ideal for various applications, including portable electronics, electric vehicles, and renewable energy systems.^49^

## 2 Experimental Section

### 2.1 Sample Preparation

#### 2.1.1 Synthesis of Ge–H and Ge–C_2_–CN Materials

Ge–H was synthesized according to previously published work.^50^ Briefly, CaGe_2_ (1 g) was crushed into approximately 5 mm crystals and then reacted with concentrated hydrochloric acid (HCl, 150 mL) at a temperature of −30 °C. The reaction was accomplished in a Schlenk tube that had been purged initially with argon to remove oxygen. The content of the Schlenk tube was periodically agitated usually once per two or 3 days for a period of 10 days, and then Ge–H was obtained by filtration, followed by successive washing steps with distilled water (5 × 200 mL) and MeOH (2 × 100 mL). The final product was then dried under vacuum and stored in an argon-filled glovebox.

The synthesis of Ge–C_2_–CN was also performed according to a previously published procedure.^34,51^ In this case, CaGe_2_ (400 mg) and 3-bromopropionitrile (BrCH_2_CH_2_CN, 5 mL) were reacted together at room temperature within a biphasic system composed of water and an alkyl halide. The separation of the phases was achieved by utilizing porous silica, which facilitated the retention of CaGe_2_ in the organic phase. After 10 days, the product was isolated by filtration. The isolated compound underwent a series of washing steps using 1 M hydrochloric acid (2 × 100 mL), acetone (2 × 100 mL), water (5 × 100 mL), and MeOH (2 × 100 mL) and finally dried in a vacuum.

#### 2.1.2 Preparation of the Ge–H and Ge–C_2_–CN Photocathodes

A circular conductive and transparent substrate with a diameter of 15 mm made by flexible poly(ethylene terephthalate) (PET)-coated indium tin oxide (ITO, ∼80 nm) and gold (Au, ∼20 nm) was chosen to drop-cast the synthesized Ge–H and Ge–C_2_–CN slurries on top of them. The purpose of the Au coating layer on PET-coated ITO was to enhance the conductivity of the substrate and ensure the ITO stability during subsequent analyses. Prior to applying the Ge–H and Ge–C_2_–CN slurries onto the PET-coated ITO/Au, 45 mg (90 wt %) of each synthesized Ge–H and Ge–C_2_–CN was mixed completely with 2.5 mg of graphene oxide (GO, 5 wt %) as a carrier for electrical charges and 2.5 mg of poly(vinylidene fluoride) [PVDF, 5 wt %, Alfa Aesar, Thermo Fisher Scientific] binder in 2 mL of *N*-Methyl-2-pyrrolidone (NMP, 99% extra pure, Acros Organics, Thermo Fisher Scientific) solvent. The mixture was then subjected to ultrasonic treatment for at least 1 h and then kept under stirring (200–250 rpm) overnight to achieve a homogeneous slurry. Following this, 10 μL of each slurry was drop-cast onto the as-prepared PET-coated ITO/Au substrate and dried in a vacuum oven set at 35 °C for a duration of 24 h. Later on, a drop of Nafion (C_7_HF_13_O_5_S.C_2_F_4_) was spread out on top of the electrode to build a stable structure during the electrochemistry test and again kept in a vacuum oven for a few hours to be dried before starting further analyses.

#### 2.1.3 Design and Fabrication of the Photo-E ZIC Cells

The photocathodes that had been prepared from the previous stage, along with a precut circular Zn foil anode with ∼1 μm thickness and 15 mm diameter, a circular Whatman glass microfiber filter paper separator (18 mm in diameter), and a 2016-type coin cell positive case containing 30 μL of 2 M ZnSO_4_·7H_2_O aqueous electrolyte, were assembled inside a printed holder. This holder was printed using polylactic acid (PLA) filament (Prusa Research, Czech Republic) with the feature of an optical window (8 mm diameter). To ensure a secure seal for the cell, a square-shaped sheet with a 1 mm thickness and a hole with an 8 mm diameter at its center was also printed and then placed over the assembled capacitor cell to provide complete sealing.

### 2.2 Characterization Methods

#### 2.2.1 Material Characterization

The crystal and phase characterization of the synthesized Ge–H and Ge–C_2_–CN materials and their slurries were identified by X-ray diffraction (XRD) at room temperature on a Bruker D8 Discoverer (Bruker, Germany) with Cu Kα radiation (λ = 0.15418 nm, *U* = 40 kV, *I* = 40 mA). The samples were deposited on a zero diffraction silicon plate and the data were scanned over the angular range 3–60° (2θ, for the synthesized materials) and 5–90° (2θ, for the slurries) with a step size of 0.02°. The collected data evaluation was performed using the software package EVA. Fourier transform infrared spectroscopy (FTIR) measurements were performed by an iS50R FTIR spectrometer (Thermo Scientific). The measurement was performed using a DLaTGS detector and KBr beam splitter in the range 4000–400 cm^–1^ at a resolution of 4 cm^–1^. FTIR spectra of the samples were measured in a KBr pellet made from 300 mg of KBr and 1 mg of the synthesized materials. Raman spectroscopy characterization on the Ge–H, Ge–C_2_–CN, and GO (conductive additive) was performed by using a Raman microscope (Renishaw, England) in backscattering geometry with a CCD detector. The Cobalt 08 narrow line width diode laser (785 nm, 120 mW) with an applied power of 0.5% and 20× magnification objective was used for this purpose. The spectra were referenced according to a laser peak position at 0 cm^–1^, and the spectrometer was referenced before the measurements by acquiring the spectrum of silicon and referenced to 521 cm^–1^. The samples were deposited on a metal plate as a powder and measured immediately after the deposition. High-resolution X-ray photoelectron spectroscopy (XPS) was performed using an ESCA Probe P spectrometer (Omicron Nanotechnology Ltd., Germany) with a monochromatic aluminum X-ray radiation source (1486.7 eV). Wide-scan surveys of all elements were performed with subsequent high-resolution scans of the C 1s, O 1s, N 1s, Cl 2p, and Ge 2p. Relative sensitivity factors were used to evaluate the element ratios from the survey spectra. The samples were placed on a conductive carrier, and an electron flood gun was used to eliminate sample charging during measurement (1–5 eV). The values were referenced to the adventitious carbon peak at 284.8 eV. A PerkinElmer Lambda 850+ spectrometer coupled with integrating sphere was used for the ultraviolet–visible (UV–vis) measurements. A tungsten lamp (250–850 nm) and 0.16 s nm^–1^ integration time were used. A quartz glass cuvette with a length of 1 cm was filled with acetonitrile suspension of Ge–H and Ge–C_2_–CN (3 mg mL^–1^) prepared before the measurement by sonication of suspension for at least 30 min. The morphology and elemental mapping of the synthesized materials, slurries, and photocathode samples after cycling stability were determined by scanning electron microscopy coupled with energy-dispersive X-ray spectroscopy (SEM/EDS, Tescan Lyra 3) and transmission electron microscopy (TEM) with a JEOL 2200 FS microscope at 200 kV and JEOL monochromated ARM200F at 200 kV. To obtain spatially resolved information about the localized electrochemical reactivity at the microscale, scanning electrochemical microscopy (SECM, Sensolytics, Germany) was employed as a powerful analytical technique. This technique could provide information about the mapping of the surface physical properties, catalytic activity on the surface, and charge transfer kinetics. The feedback mode was used to map the surface and the conductivity of the Ge–H and Ge–C_2_–CN suspensions used as the electrode materials in the photo-E device. In the four-dimensional (4D) array scan, the ultramicroelectrode (UME) approaches the surface at each point from the bulk solution above the specific area of the sample and observes the change in the Faraday current. The change can be caused by the reactivity, roughness of the sample, distance between the electrode and the surface, size of the UME, and geometry of the diffusion region around it. A Pt UME with a 5 μm diameter as a tip working electrode was employed to scan the surface of materials with a chosen specific area of 100 μm^2^ using 2.5 μm increments in the *x* and *y* directions. A spiral-shaped Pt wire also was used as the counter electrode. Around 10 μL of each slurry was drop-cast on precleaned silicon wafers and dried in a vacuum oven before doing the tests. The SECM characterization was done inside the potassium ferrocyanide redox system K_4_[Fe(CN)_6_] solution including 10 mmol L^–1^ ferrocyanide [Fe(CN)_6_]^4–^ as a redox mediator and 0.1 mol L^–1^ potassium chloride (KCl) as a supportive electrolyte. The UME potential was set at *E* = 0.5 V versus the Ag/AgCl reference electrode to drive the redox reaction Fe(CN)_6_^4–^ → Fe(CN)_6_^3–^ + e^–^ and to reach a steady state.

#### 2.2.2 Electrochemical Characterizations

Electrochemical analysis of the photo-E ZICs was performed by an Autolab PGSTAT 204 (NOVA, Utrecht, Netherlands) under dark and illuminated conditions. The electrochemical performance was assessed by using a range of techniques including cyclic voltammetry (CV), galvanostatic charge–discharge (CD), electrochemical impedance spectroscopy (EIS), and chronoamperometry. The CV and CD measurements were analyzed at different scan rates, ranging from 5 to 300 mV s^–1^, and specific currents from 5 to 60 mA g^–1^. These measurements were carried out within the optimized voltage window of 0.2–1.0 V. EIS measurements were conducted using an open circuit potential at a voltage amplitude of 10 mV in the frequency range of 10 mHz to 100 kHz, under dark and illuminated conditions. Chronoamperometry analyses were performed at zero applied voltage using different LED sources of 435 nm (violet), 533 nm (green), and 630 nm (red) wavelengths under 50 and 100 mW cm^–2^ illumination to evaluate the photocurrent responsivity of the samples. Furthermore, three-electrode systems also were established to evaluate the capacitance and photocurrent responsivity of the samples using the same potentiostat. To do so, 10 μL of each slurry was drop-cast on PET-coated ITO/Au substrates serving as the working electrode, a platinum (Pt) wire functioned as the counter electrode, and a Hg(l)/Hg_2_Cl_2_(s)/KCl(sat) electrode was employed as the reference electrode. The CV scans within this three-electrode system were conducted over a voltage window from 0.0 to 0.5 V, and scan rates ranged from 5 to 100 mV S^1–^. Photocurrent responsivity of the samples at zero applied voltage under illumination by a 435 nm wavelength LED source was tested by chronoamperometry characterization. Additionally, the distinct responses of the samples to illumination were explored using linear sweep voltammetry (LSV) at a scan rate of 5 mV s^–1^ under dark and illumination (100 mW cm^–2^) conditions.

#### 2.2.3 Density Functional Theory Calculations

Density functional theory (DFT) calculations were performed with the Quantum Espresso 7.1 software package.^52−54^ It is conducted with a periodic unit cell consisting of eight Ge atoms terminated with eight H atoms in the case of Ge–H and six H atoms and two CH_2_CH_2_CN groups in Ge–C_2_–CN. This composition is very close to our experimental composition and therefore was used in the modeling. The structures were relaxed to obtain the minimum using the vdW-DF3-opt1 DFT functional,^55^ within the hexagonal unit cell with *a* parameter 8.00 Å and *c* parameter 20.0 Å for Ge–H and 8.10 and 33.0 Å for Ge–C_2_–CN, respectively. The *c* parameters were chosen to contain enough vacuum to neglect any interaction between the layers. Further calculation parameters were the following: plane wave cutoff energy *E*_cut_ = 100 Ry, energy convergence criterion <1.0 × 10^–8^ Ry, and *k* point sampling 4 × 4 × 1. Scalar relativistic norm-conserving pseudopotentials^56^ were used for all atoms. The HSE06 functional^57^ was used to calculate band gaps.

## 3 Results and Discussion

**Figure 1:**
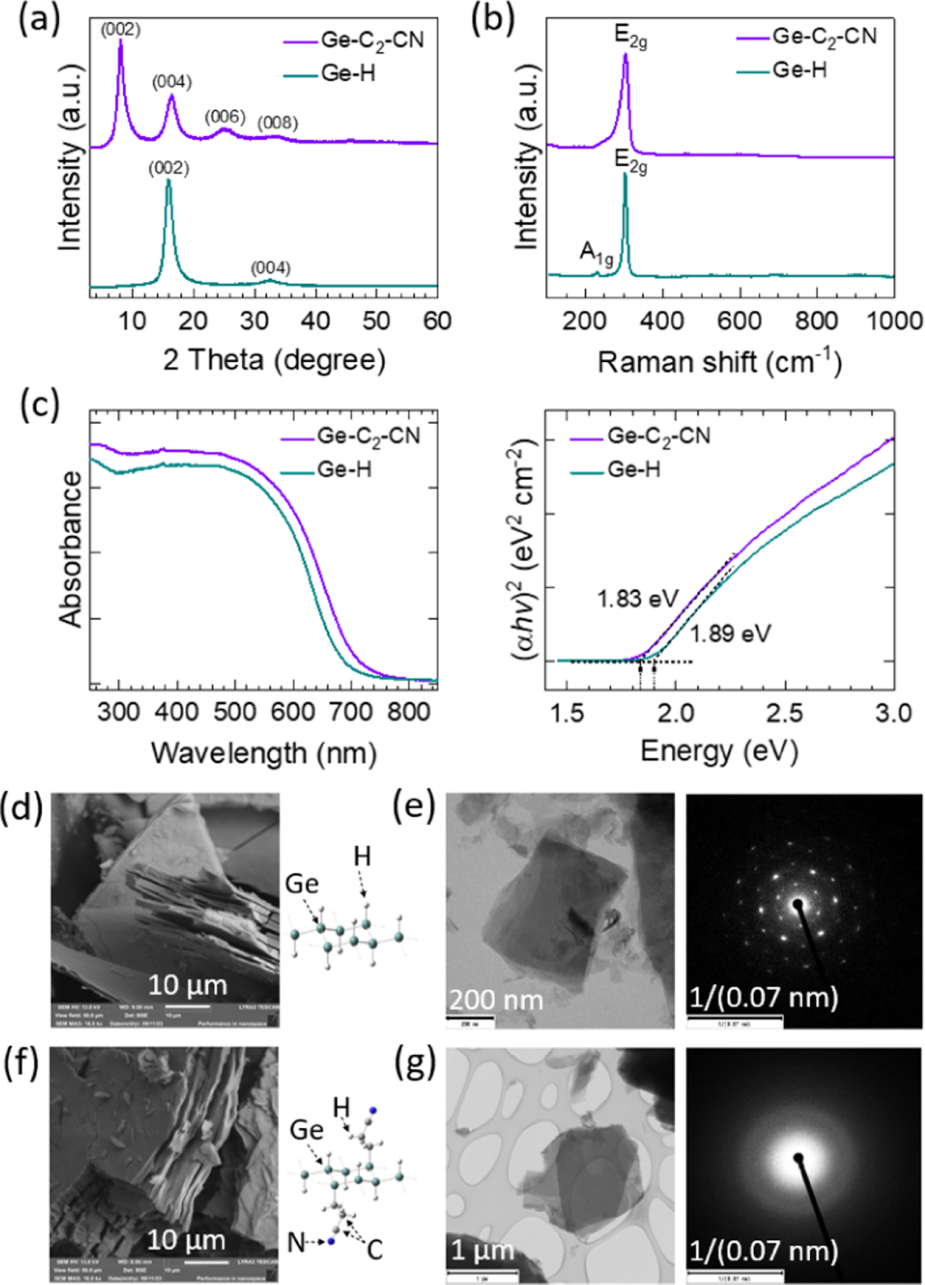
(a) XRD pattern, (b) Raman spectrum, (c) UV–vis and evaluated band gap, (d, e) SEM, TEM, and electron diffraction pattern of Ge–H powder and (f, g) SEM, TEM, and electron diffraction pattern of Ge–C_2_–CN powder.

Figure 1a shows the crystal phases of the Ge–H and Ge–C_2_–CN samples evaluated by XRD. It can confirm the successful exfoliation of CaGe_2_ into layered materials. The structure of Ge–H can be fitted into a hexagonal 2H unit cell with two Ge–H layers per cell.^50^ It should be noted that due to the nature of 2D materials, the synthesized materials have variable interlayer distance, making the corresponding reflections broad, and further refinement via Rietveld analysis is impossible. The first reflection (002) was used for the evaluation of the interlayer distance. It was found that Ge–H has an interlayer distance of 5.615 Å and that value was 10.983 Å for the Ge–C_2_–CN sample. This difference in interlayer spacing is relevant to the length of the cyanoethyl alkyl chain. Further analysis of the samples by FTIR (Figure S1) also acknowledged the chemical transformation of CaGe_2_ into modified Ge–H and Ge–C_2_–CN materials. The vibration bonds of Ge–H are observed at 1994, 578, and 477 cm^–1^. The Ge–H sheets are also edge-terminated by GeH_2_ units as evident from vibrations at 824 and 766 cm^–1^. The Ge–C_2_–CN material showed several vibrations related to C–H groups (2916, 1412, 1151, 1003, ∼800 cm^–1^) and characteristic CN group vibration (2246 cm^–1^). Incomplete modification by the alkyl chain due to its higher steric demand that hydrogen resulted in the termination of a portion of germanium atoms instead of the alkyl chain is evident from Ge–H vibration at 1980 cm^–1^. Besides, corresponding Ge–C and Ge–H vibrations were also identified in the region of ∼400–600 cm^–1^. In addition, a small portion of absorbed water was found in both materials obvious by vibration at ∼1610 cm^–1^.

Raman analysis (Figure 1b) also confirms Ge–H exfoliation and conservation of Ge–H skeleton during the exfoliation, as evident from the E_2g_ peak at 303 cm^–1^ for Ge–H and 304 cm^–1^ for Ge–C_2_–CN. In the case of the Ge–H sample, a weak A_1g_ mode was exhibited at 231 cm^–1^. The surface composition of the samples was studied by XPS (Figure S2). It is noted that the presented binding energies have only informative character due to the semiconducting properties of materials.^58^ According to the survey spectrum, the Ge–H material is composed of germanium (95.5%) and chlorine (4.5%). Carbon and oxygen are present only as adventitious surface contamination. The Ge 2p region was fitted with only one peak corresponding to the Ge–H mode (1217.21 eV). Chlorine is probably present in the form of Ge–Cl (Ge–Cl 2p_3/2_ 198.90 eV, Ge–Cl 2p_1/2_ 200.49 eV) due to the initial reaction with HCl during the synthesis and formation of some Ge–Cl. That mode was not included in the Ge 2p region because of its similarity to the binding energy of Ge–H. The Ge–C_2_–CN material was composed of germanium, carbon, nitrogen, and oxygen, without detection of bromine in the XPS spectrum. Based on the survey spectrum and corresponding high-resolution spectra, the simplified formula of this material is approximately Ge_6_H_4_O_0.2_(C_2_H_4_CN)_2_. The Ge 2p region was fitted with two curves indicating Ge–C and Ge–H modes (1216.98 eV, 96.18%) and oxidized germanium (Ge–O, 1220.81 eV, 3.82%). Successful covalent modification with the cyanoethyl alkyl chain is evident from the C 1s spectrum with three modes corresponding to Ge–C (282.80 eV, 14%), the CN group (286.84 eV, 12.78%), and C–C with adventitious carbon (284.77 eV, 73.22%). The CN group was also confirmed by the N 1s spectrum with a binding energy of 400.05 eV.

According to the O 1s spectrum, the majority of oxygen was identified as adventitious contamination (532.18 eV, 88.43%), and the rest corresponded to oxidized germanium (529.57 eV, 11.57%).^59^ The optical band gap of the materials was estimated based on UV–vis spectra and the Tauc plot approach using the formula for direct transition.^60^ It can be seen that the band gap value of Ge–H is estimated to be 1.89 eV, while this value was reduced to 1.83 eV for Ge–C_2_–CN (Figure 1c). According to DFT calculations, both materials have a direct band gap. The band gap was estimated to be 2.04 eV for Ge–H and 1.87 eV for Ge–C_2_–CN. However, it should be noted that spin–orbit coupling was not taken into account with the approximate value of 0.1–0.2 eV.^50^ Therefore, the calculated values match the observed band gaps very well. Figure 1d–g shows the morphology of the synthesized Ge–H and Ge–C_2_–CN samples, respectively, which were characterized by SEM, TEM, and electron diffraction patterns. It can be seen that the electron diffraction pattern of the Ge–C_2_–CN sample does not show any crystallinity. This effect could be attributed to the high degree of functionalization of germanane with carbon and nitrogen. Figure S3 also shows the SEM and EDS images of the synthesized Ge–H and Ge–C_2_–CN powders. The results confirm a layered structure for both samples including the elementals that already were confirmed by XPS.

**Figure 2:**
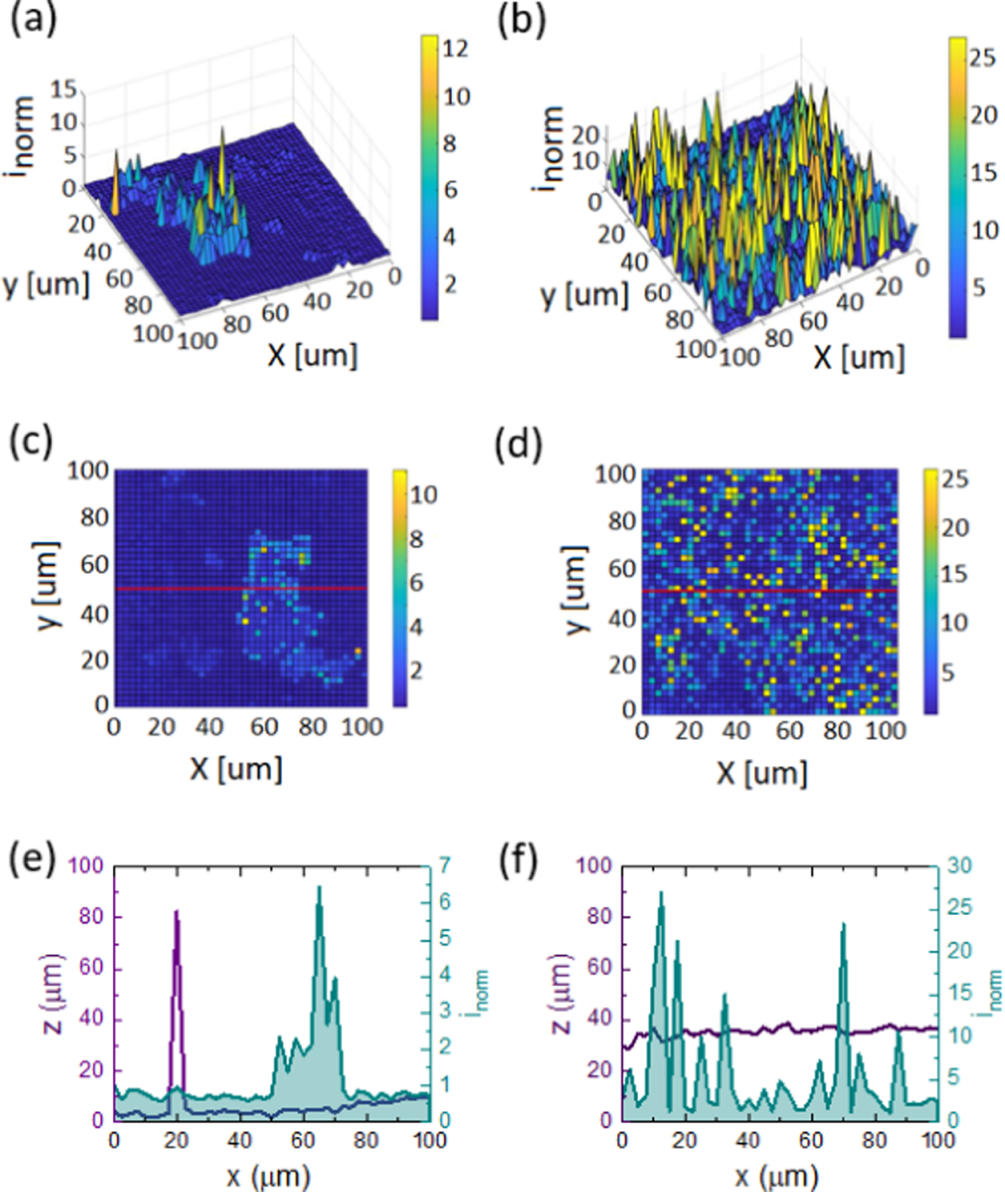
(a, b) 3D and (c, d) 2D topographical mapping catalytic activities of drop-cast Ge–H and Ge–C_2_–CN slurries and roughness (*z*) and normalized tip current (*i*_norm_) values of the red line for (e) Ge–H and (f) Ge–C_2_–CN samples corresponding to the 2D topographical images.

Figure S4 illustrates the SEM and EDS images of Ge–H and Ge–C_2_–CN slurries produced by these 2D materials. Both of the samples have carbon in addition to their chemicals. The extra carbon belongs to GO as a conductive-binding matrix, which was added to the Ge–H and Ge–C_2_–CN to form slurry. Raman spectra and XRD patterns in Figure S5 illustrate the GO phase that was chosen in this study and the slurries deposited on top of PET-coated ITO substrates, respectively. 3D and 2D topographical mappings of current in the surface vicinity of both drop-cast slurries are exhibited in Figure 2a–d, respectively. The roughness (*z*) and normalized tip current (*i*_norm_) values of one scanned line also are shown in Figure 2e,f corresponding to the 2D topographical images for Ge–H and Ge–C_2_–CN slurries, respectively. The evaluated current values were normalized to be able to compare the performance of the tested materials from SECM. The normalization was done using current (*i*_bulk_) in the electrolyte high above the surface for each sample, where the current values are constantly reaching the steady state. The measured current using the feedback mode in our case is influenced by the roughness (geometry) and physical properties (i.e., conductivity) of the material. When the UME is in the adjacent surface of the insulating material, the hemispherical diffusion layer is blocked, resulting in a decrease in current. When the UME is approaching a conductive (or electrochemically active) surface, the current increases despite the blocked hemispherical diffusion layer due to the regeneration of the mediator on the surface of conductive species.^61^ As can be seen from the peak at 20 μm in Figure 2e, the contribution of the roughness to the overall measured current is negligible. Thus, the obtained positive feedback current, for example, in between 50 and 75 μm of the Ge–H sample, in the *x* direction is caused by the conductive properties of Ge–H and Ge–C_2_CN samples. As a result, the *i*_norm_ value of the dried slurry made by the Ge–C_2_–CN sample (Figure 2f) is almost 2 times greater than that of Ge–H (Figure 2e). This enhancement is due to the relatively faster regeneration of the Fe(CN)_6_^3–^ + e^–^ → Fe(CN)_6_^4–^ redox reaction on the Ge–C_2_–CN surface, which is caused by superior conductivity and/or electrochemical activity in this sample. This enhanced contribution is due to the additional carbon and nitrogen elements in comparison to Ge–H which can tailor the electrical properties of the Ge–C_2_–CN material.

**Figure 3:**
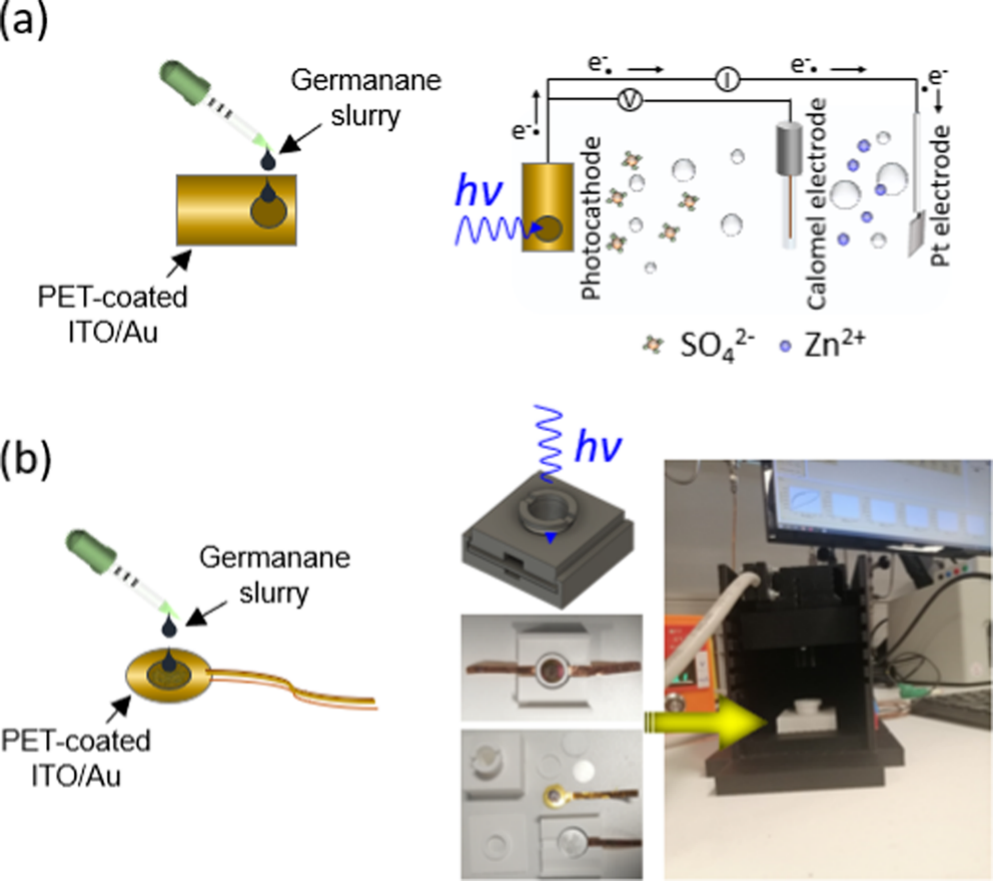
Schematic illustrations of (a) three-electrode and (b) two-electrode cells set up in this study.

**Figure 4:**
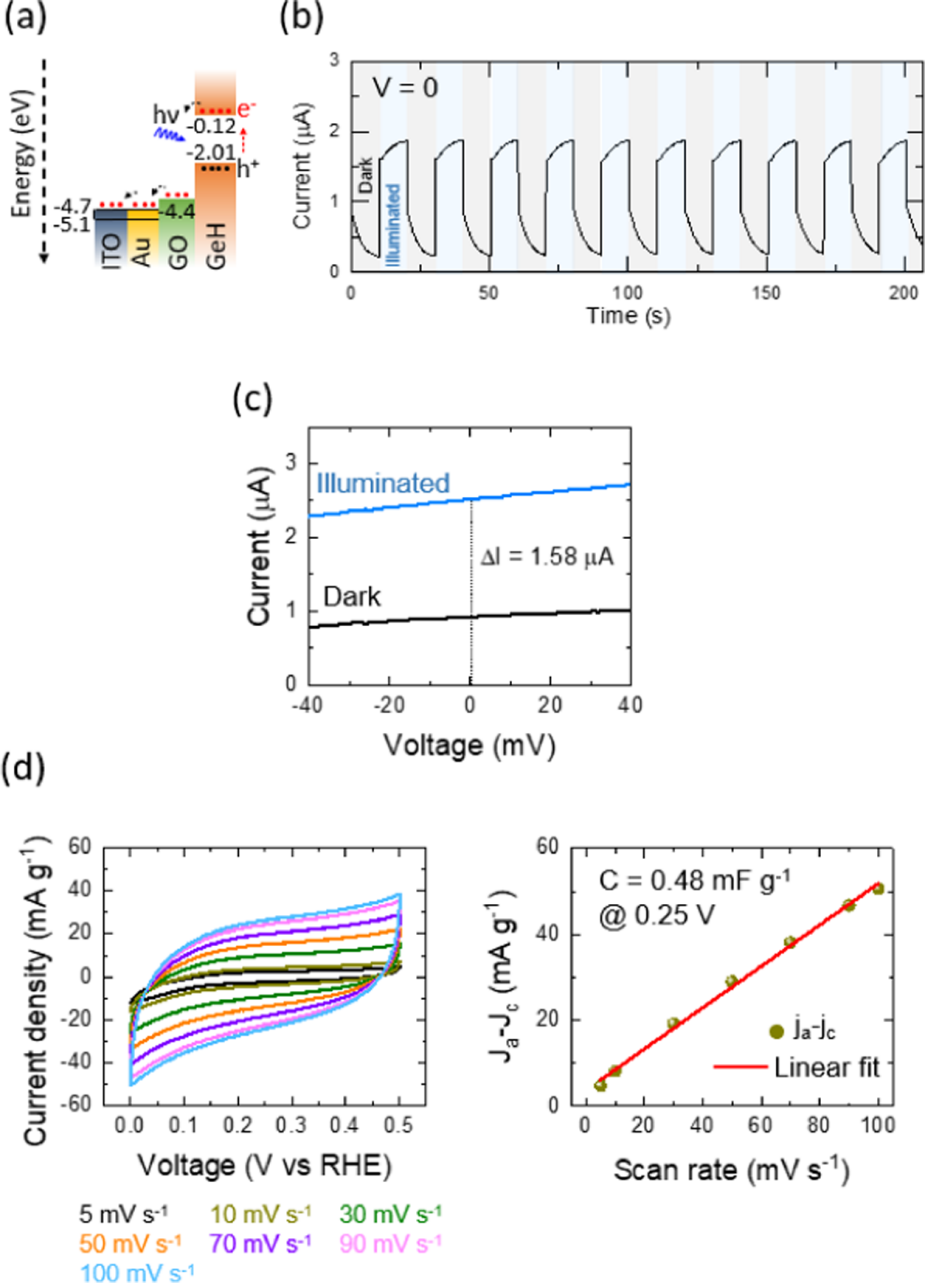
(a) Schematic illustration of the energy band diagram and energetically desirable pathways of the Ge–H photocathodes constructed on the PET-coated ITO/Au substrate. (b) Current–time (repeated dark and illumination at 0 applied voltage), (c) current–voltage (dark and illumination, at a scan rate of 5 mV s^–1^), (d) cyclic voltammograms at different scan rates and determination of double-layer capacitance plots of the Ge–H. Note: all examined tests were done under dark and illumination conditions by an LED with λ = 435 nm and an intensity of 100 mW cm^–2^.

**Figure 5:**
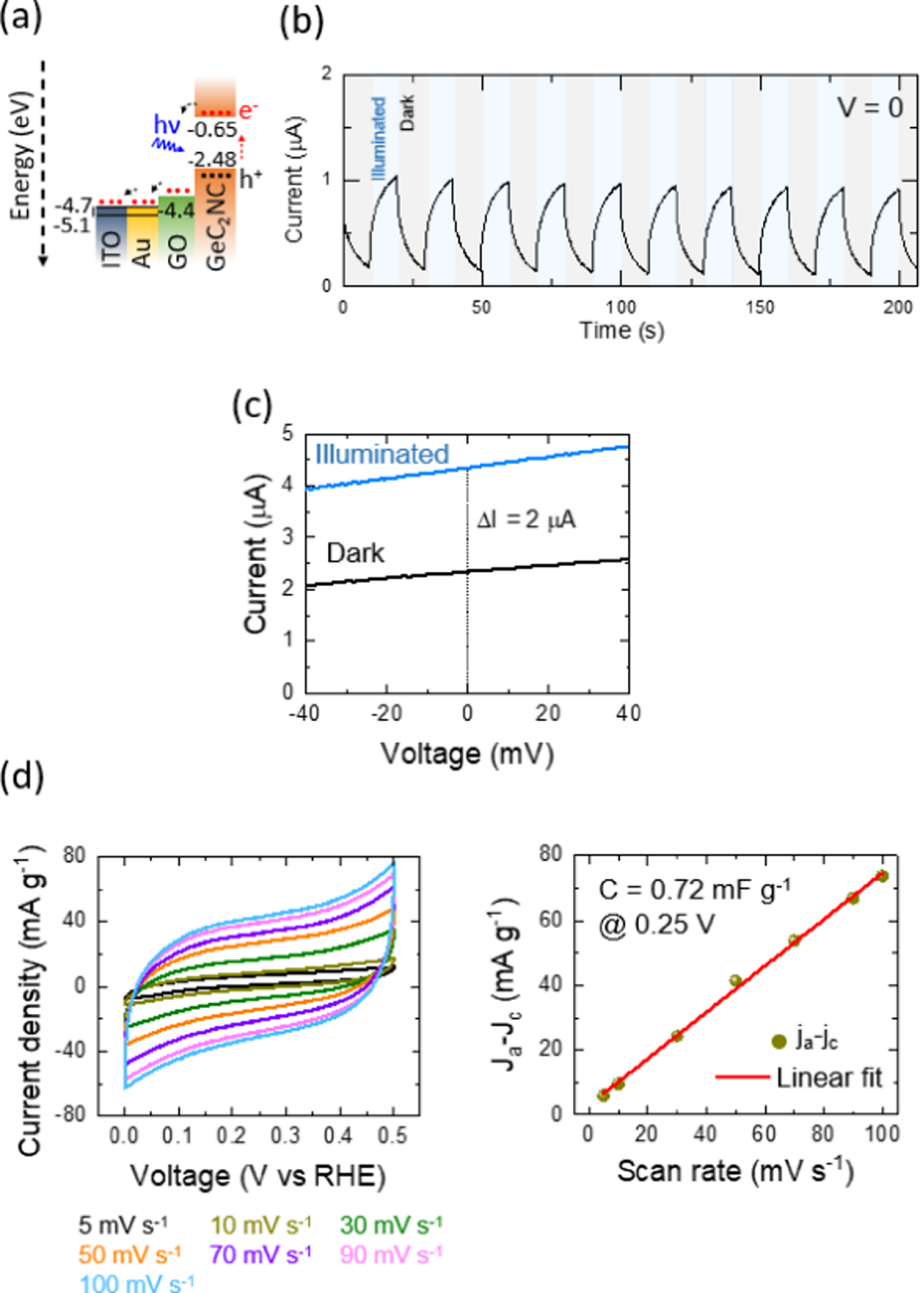
(a) Schematic illustration of the energy band diagram and energetically desirable pathways of the Ge–C_2_–CN photocathodes constructed on the PET-coated ITO/Au substrate. (b) Current–time (repeated dark and illumination at 0 applied voltage), (c) current–voltage (dark and illumination, at a scan rate of 5 mV s^–1^), (d) cyclic voltammograms at different scan rates and determination of double-layer capacitance plots of the Ge–C_2_–CN. Note: all examined tests were done under dark and illumination conditions by an LED with λ = 435 nm and an intensity of 100 mW cm^–2^.

The schemes of three-electrode and two-electrode cells set up in this study are illustrated in Figure 3a,b. As configured in Figure 3a, photogenerated electrons after irradiation move from the photocathode and accumulate on the Pt counter electrode with Zn^2+^ cation attraction from the ionized zinc sulfate electrolyte. At the same time, the photogenerated holes on the photocathode tend to absorb the SO_4_^2–^ anions. The same mechanism occurs in the two-electrode system where Zn^2+^ cations and SO_4_^2–^ anions attract the Zn anode and photocathode to store electric charges. To recognize the optoelectronic properties and electrochemical behavior of the photocathodes fabricated by Ge–H and Ge–C_2_–CN, a self-powered photodetector device was set up in a beaker by a three-electrode cell. The optoelectronic efficiency of the photocathodes was determined by an LED with λ = 435 nm and an intensity of 100 mW cm^–2^, in this section of the work. Figures 4a and 5a illustrate the energy band diagram and energetically desirable pathways of the Ge–H and Ge–C_2_–CN photocathodes after illumination. It is a widely recognized phenomenon that when a material is exposed to light with a higher energy level than the material band gap, it results in the generation of photoelectrons and holes due to the photoelectric effect.^62^ Therefore, since the Ge–H and Ge–C_2_–CN materials exhibit lower band gap values (1.89 eV for Ge–H and 1.83 eV for Ge–C_2_–CN) than the energy of the incident light (λ = 435 nm with ∼2.85 eV), this facilitates the migration of photoelectrons from those materials toward the PET-coated ITO/Au substrate through GO and circulate in the system.

The current–time plots under periodic illumination (10 s off and 10 s on) and zero applied voltage were carried out on the samples to evaluate their photocurrent behaviors (Figures 4b and 5b). It can be seen that the current signal rises instantly under illumination and drops when the light is turned off. The photocurrent intensity in the Ge–H photocathode reached ∼2 μA, while that value is ∼1 μA for the Ge–C_2_–CN photocathode. This incident is most probably due to the higher conductivity of the Ge–C_2_–CN sample in the presence of carbon and nitrogen. To evaluate the photocurrent behavior further, the LSV characteristics of the photocathodes were executed in dark and illuminated conditions (Figures 4c and 5c). The results show a higher current under illumination even at the applied voltage of 0 V. These treatments suggest ideal photosensitivity of the Ge–H and Ge–C_2_–CN photocathodes with susceptibility to electron and hole separation and generate a higher photocurrent under illumination than under dark conditions. It is observed from LSV characterization that the current response with the Ge–C_2_–CN photocathode is higher than the current response with the Ge–H photocathode, while this effect was opposite with chronoamperometry. This can be argued as the reason that the current response in chronoamperometry is expected to be more stable and constant over time, while the current response in LSV is expected to be more variable and dependent on the rate of electrochemical processes occurring at the photoelectrode surface. The applied voltage in chronoamperometry is constant during the whole of the experimental procedure and this makes the material stable during this test. However, the applied voltage is variable in the LSV procedure, and even at 0 V, there already would be an electrochemical process on the materials at a voltage range of lower and higher zero voltage that makes the electrode unstable. Therefore, the different current responses observed in these two techniques can be attributed to the different measurement parameters and electrochemical processes on the photoelectrode materials. The energy storage potential of the Ge–H and Ge–C_2_–CN photocathodes also was evaluated by the three-electrode cell using the CV responses at different scan rates over the working voltage of 0–0.5 V (Figures 4d and 5d). The choice of that low working voltage range was due to avoiding any gas formation. It is observed that there is a difference in CV responses between Ge–H and Ge–C_2_–CN. The current density and double-layer capacitance reach 80 mA g^–1^ (at 100 mV s^–1^) and 0.72 mF g^–1^ (at 0.25 V) in Ge–C_2_–CN; however, those values are 40 mA g^–1^ (at 100 mV s^–1^) and 0.48 mF g^–1^ (at 0.25 V) in Ge–H. These differences also could be due to the presence of carbon and nitrogen elements with the potential to enhance current density and capacitance.

**Figure 6:**
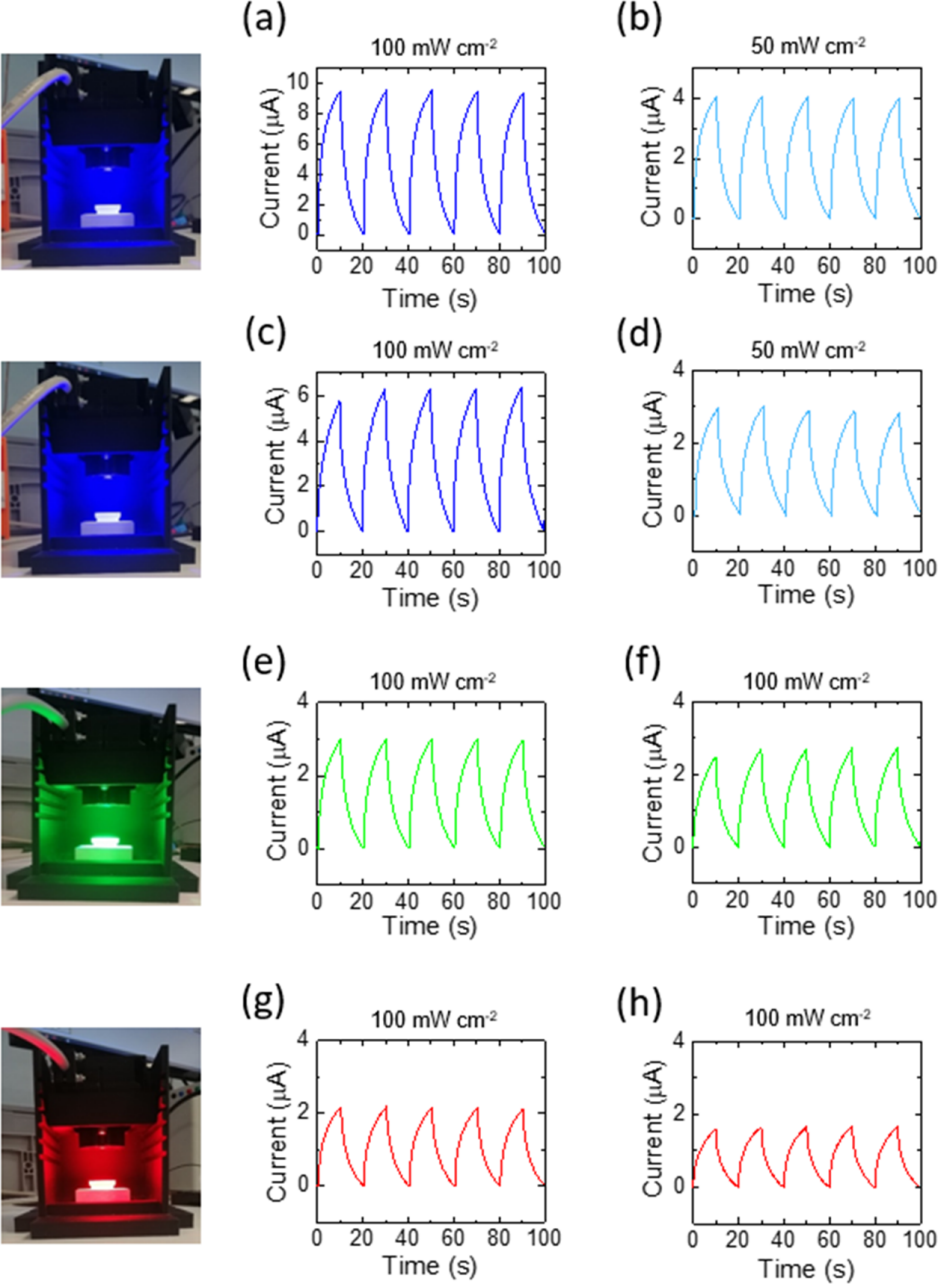
Cyclic illumination and dark-current responses of the (a, b) Ge–H and (c, d) Ge–C_2_–CN photo-E ZICs using an LED with λ = 435 nm (blue, 100, and 50 mW cm^–2^), (e) Ge–H and (f) Ge–C_2_–CN photo-E ZICs using an LED with λ = 533 nm (green, 100 mW cm^–2^), and (g) Ge–H and (h) Ge–C_2_–CN photo-E ZICs using an LED with λ = 630 nm (red, 100 mW cm^–2^) at zero applied voltage (0 V). Note: the *y*-axis is equal to Δ*I* = *I*_light_ – *I*_dark_, where they stand for the currents in illumination (*I*_light_) and dark (*I*_dark_) conditions.

Photo- and dark-current efficiency of the Ge–H and Ge–C_2_–CN photo-E ZICs was also analyzed against the Zn anode by 2016-type coin cells inside the optically printed holder. This characterization was done by light-dependent chronoamperometry in dark and illuminated conditions, and three different LEDs of λ = 435 nm (50 and 100 mW cm^–2^), λ = 533 nm (100 mW cm^–2^), and λ = 630 nm (100 mW cm^–2^) at 0 applied voltage were employed for this purpose (Figure 6).

It is observed in Figure 6 that the maximum current response of the photocurrent value, which is obtained by Δ*I* = *I*_light_ – *I*_dark_, relates to the Ge–H photo-E ZIC under 435 nm LED and 100 mW cm^–2^ intensity. However, it reduced by almost half when the light intensity decreased to 50 mW cm^–2^. The value of photocurrents for Ge–C_2_–CN photo-E ZIC with the same examined light and intensities was less than that of the Ge–H photo-E ZIC. These results are in line with the previous photocurrent characterization in the three-electrode system. It is also identified that the photocurrents of both photo-E ZICs reduced even further when they were assessed with λ = 533 nm and λ = 630 nm at the intensity of 100 mW cm^–2^. These results are sensible because of the greater energy of 435 nm LED light (∼2.85 eV) compared to the other 533 nm (∼2.33 eV) and 630 nm (∼1.97 eV) LED lights. The higher energy of the 435 nm LED light can deliver more energy to the photocathodes and enhance the formation of photogenerated electrons that pass from the photocathode to the Zn anode.

**Figure 7:**
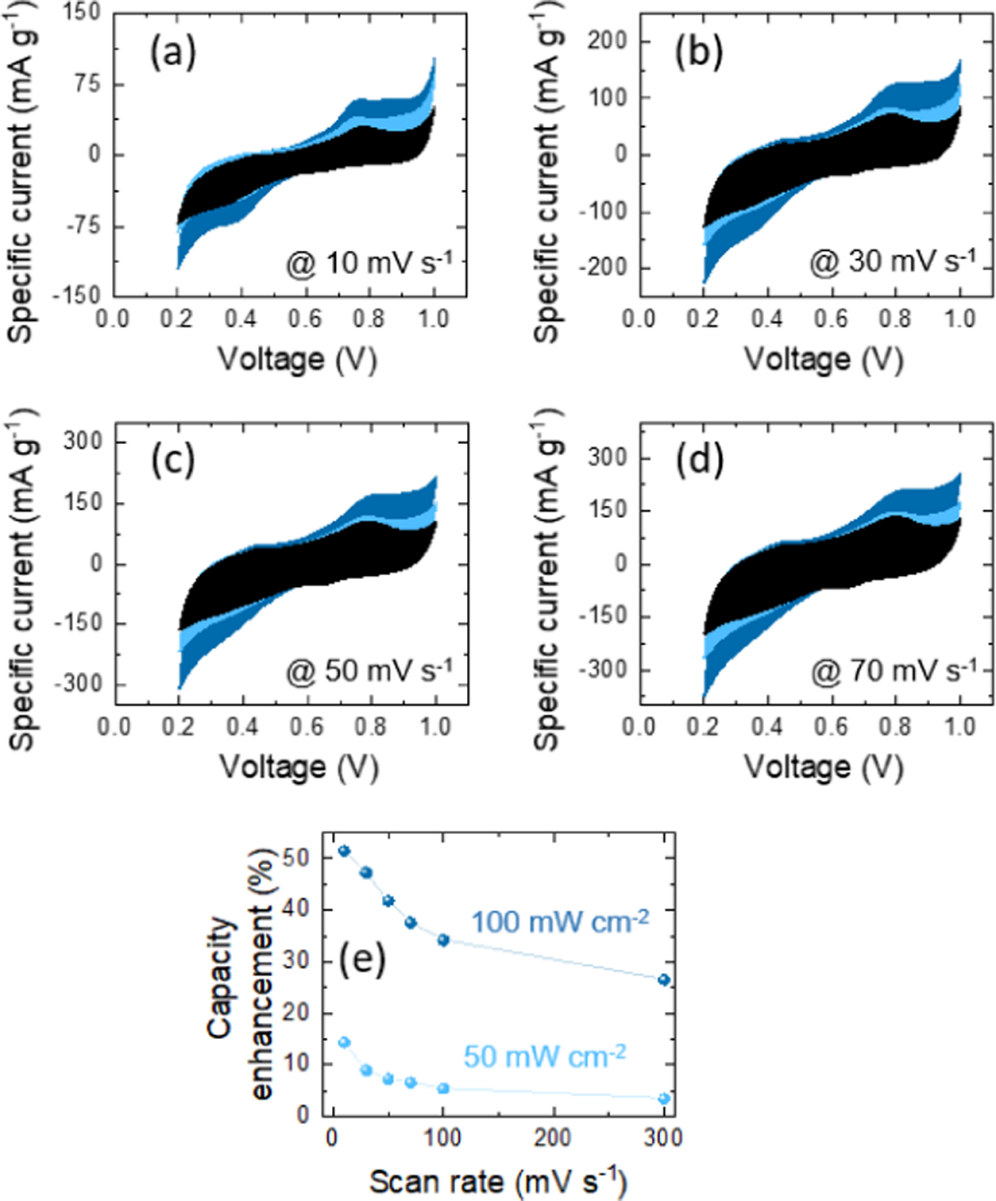
(a–d) Comparative CV analyses of Ge–H photo-E ZICs at different scan rates under dark and illumination with λ = 435 nm (light blue intensity is 50 mW cm^–2^ and dark blue intensity is 100 mW cm^–2^). (e) Diagram of the capacity enhancement versus different scan rates for Ge–H.

**Figure 8:**
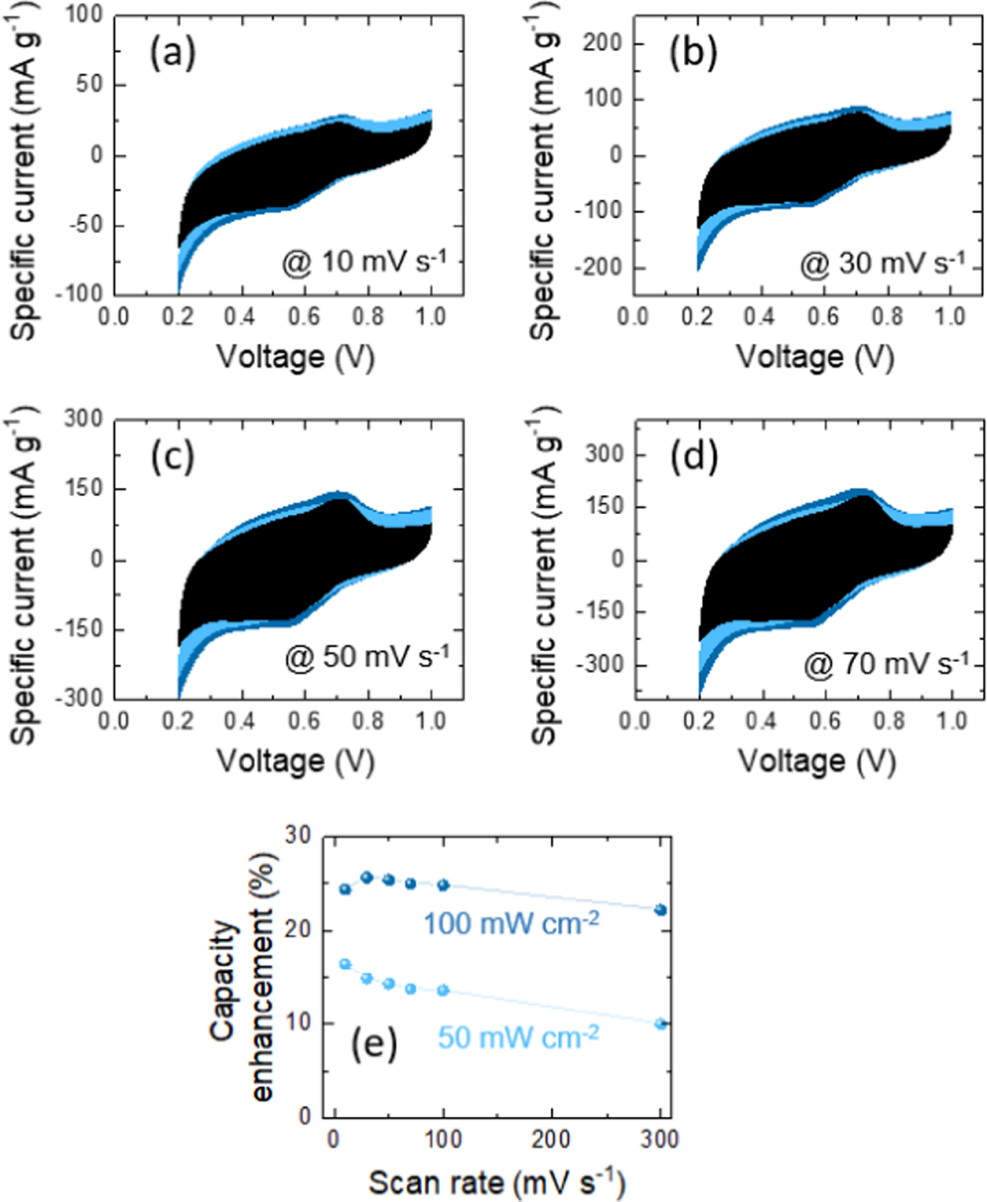
(a–d) Comparative CV analyses of Ge–C_2_–CN photo-E ZICs at different scan rates under dark and illumination with λ = 435 nm (light blue intensity is 50 mW cm^–2^ and dark blue intensity is 100 mW cm^–2^). (e) Diagram of the capacity enhancement versus different scan rates for Ge–C_2_–CN.

The electrochemical performances of the photo-E ZICs were conducted by cyclic voltammetry (CV) and galvanostatic charge–discharge (CD). Prior to starting analyses, the CV range was optimized to have valid values and to stay away from any gas formation and dehydration of SO_4_^2–^ anions and Zn^2+^ cations. Figure S6 shows the range of the initial applied voltage and the optimized range inside the dashed red border. Thus, the CV analyses were performed over the optimized voltage window of 0.2 to 1 V at different scan rates of 10, 30, 50, 70, 100, and 300 mV s^–1^ (Figures 7, 8 and S7). Nevertheless, those responses are distorted a little under illumination specifically at the higher light intensity around 0.2 and 1 V which could probably be attributed to the photocatalytic decomposition of the aqueous ZnSO_4_ electrolyte. It can be seen from the CV curves that their shapes are distinct from the common rectangular shape of the electric double-layer capacitance. This indicates that the fabricated photo-E ZICs by Ge–H and Ge–C_2_–CN materials possess pseudocapacitive behavior with intercalation and partial oxidation–reduction (redox), which is a battery-like electrochemical energy storage mechanism. However, their action is similar to a supercapacitor electrode with rapid reaction kinetics. The redox reaction behavior in these hybrid photo-E ZICs is primarily due to the reversible redox reaction that takes place at the Zn anode during charge and discharge cycles. During the charging process (oxidation), Zn ions are electrochemically stripped from the anode material and transferred into the electrolyte as Zn^2+^, releasing electrons (2e^–^) in the process. Meanwhile, the cathode does not undergo a conventional redox reaction but rather adsorbs SO_4_^–2^ ions from the electrolyte, with a role in storing energy electrostatically. During the discharge process (reduction), the reverse redox reaction occurs with a reduction reaction in the plating of Zn^2+^ from the electrolyte onto the anode material. The cathode at the same time releases the stored SO_4_^–2^ ions back into the electrolyte with the completion of the discharge cycle process. These redox reactions and electrostatic behavior of the cathode, as well as photogenerated electrons and holes all contribute to the energy storage capabilities of photo-E ZICs and make them a promising hybrid energy storage device with a balance of high power and reasonable energy density.

Both Ge–H and Ge–C_2_–CN efficiently promote the charge storage performance under illumination. The capacitance enhancements of the photo-E ZICs could be evaluated by the formula of , where *C*_light_ and *C*_dark_ are specific capacitances in illumination and dark conditions at the same scan rate, respectively.^63^ These enhancements in capacitances are attributed to the high separation of photogenerated electrons and holes, which remarkably surplus the conductivity of the electrode materials and considerably attend in charge transportation density and storage during the electrolytic procedure. The graphs of capacity enhancement versus different scan rates are provided in Figures 7e and 8e. The highest capacitance enhancements of ∼52% for Ge–H and ∼26% for Ge–C_2_–CN at a scan rate of 10 mV s^–1^ under 100 mW cm^–2^ illumination were obtained. The lower capacitance enhancement in Ge–C_2_–CN compared with Ge–H is probably due to a mismatch in the band gap value as the same trend was already observed in photocurrent responses by photodetector evaluation. These figures also indicate the role of light intensity, where the capacity is reduced as the light intensity declines to a lower value.

**Figure 9:**
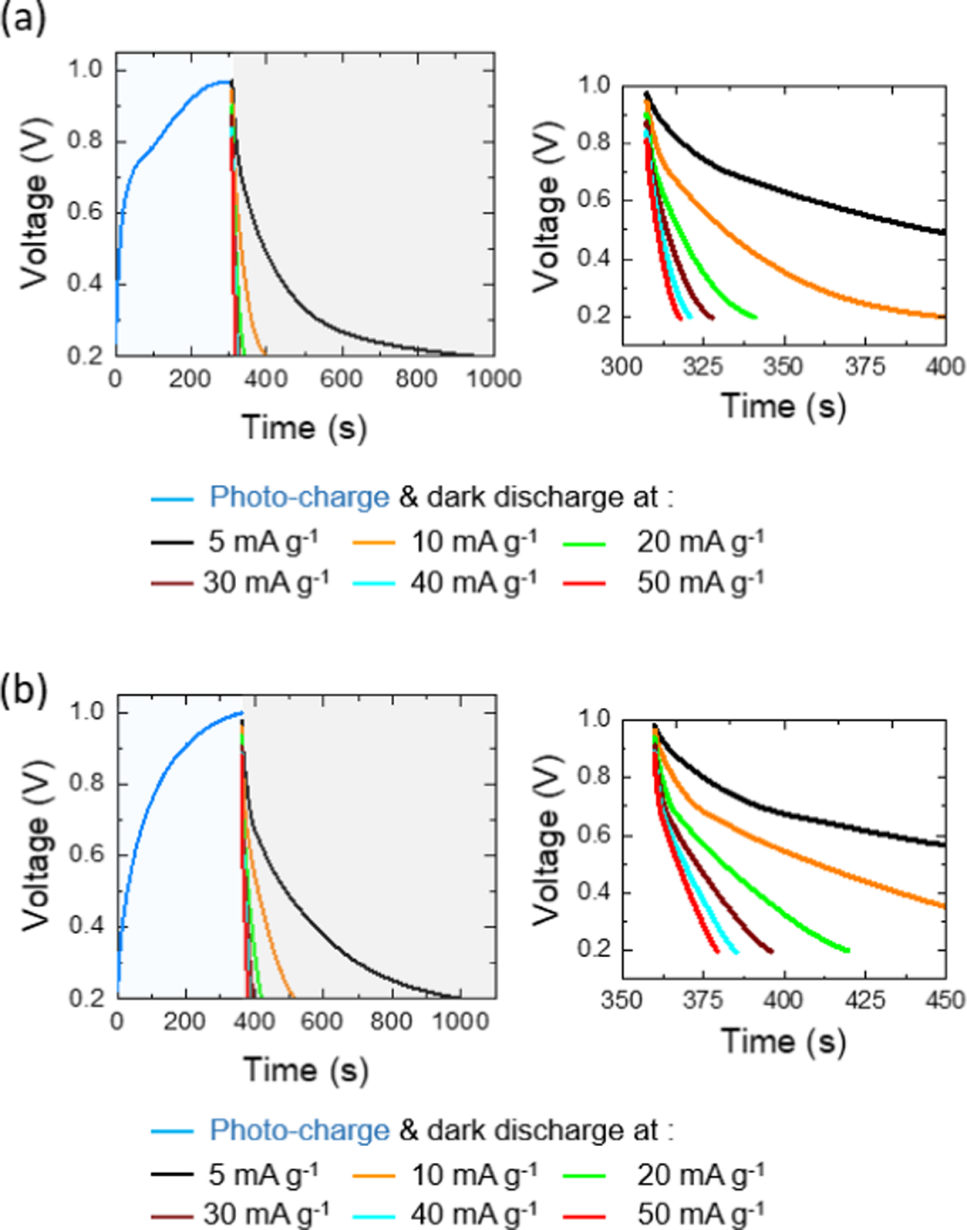
Photocharged (λ = 435 nm, 100 mW cm^–2^, at 10 mA g^–1^) and dark-discharged cycles at different specific current rates for (a) Ge–H and (b) Ge–C_2_–CN photo-E ZICs.

**Figure 10:**
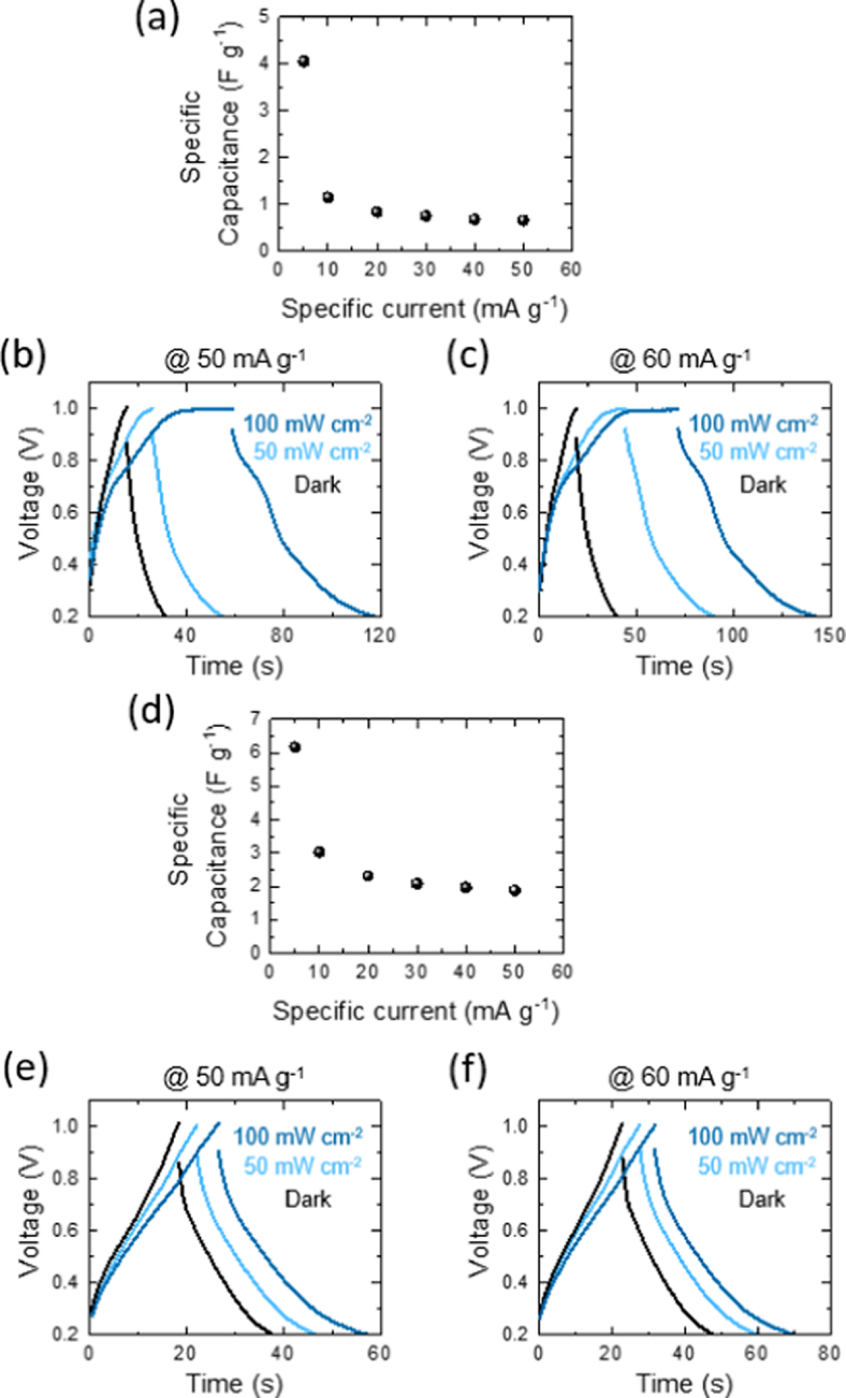
Corresponding capacity enhancements under the evaluated galvanostatic specific current rates for (a) Ge–H and (d) Ge–C_2_–CN photo-E ZICs. Comparative specific capacitances of (b, c) Ge–H and (e, f) Ge–C_2_–CN photo-E ZICs at different specific currents, CD curves at 50 and 60 mA g^–1^ in the dark, and illumination with the intensities of 50 and 100 mW cm^–2^.

In the subsequent step, photocharging and discharge strengths of the photo-E ZICs were evaluated under illumination (50 and 100 mW cm^–2^) and dark (at specific current rates of 5, 10, 20, 30, 40, 50, 60 mA g^–1^) conditions (Figures 9 and 10). Initially, as shown in Figure 9a,b, both of the photo-E ZICs were photocharged with λ = 435 nm at an intensity of 100 mW cm^–2^, a low specific current of 10 mA g^–1^, and a cutoff voltage of 1.0 V. Following this, the cells were discharged by galvanostatic at different specific current rates. It can be seen that the dark-discharge time is higher than the photocharge time at a low specific current rate of 5 mA g^–1^. This effect could be attributed to some factors such as the following: (i) The rate at which charges are generated during the photocharge process may be higher than the rate at which charges are lost during the dark-discharge process. Thus, it takes longer for the stored charges to dissipate in the dark discharge. (ii) The recombination rate of electrons and holes within the capacitor material might be relatively low in dark conditions. This will contribute to a longer dark-discharge time in a low specific current rate. (iii) The semiconductor material used to generate charges upon illumination may be favorable characteristics for charge generation, but its dark conductivity or leakage characteristics may not be as optimized in a low specific current rate. (iv) Germanane-based materials used in photoenhanced capacitors may possess trap states where charges can get trapped temporarily. These trapped charges can affect the discharge time, particularly in the dark, when there is no continuous generation of charges. (v) At low scan rates, the charging and discharging processes occur more slowly, giving more time for any recombination or leakage processes to take place, leading to a longer dark-discharge time.

On the contrary, at the higher specific current rates, the discharging time process in dark conditions is quicker than the photocharge time. This effect may be due to the optimization of the photo-E capacitor designed for high scan rates for quick response to external stimuli. The rate optimization can result in efficient charging during illumination and faster discharge upon transition to dark conditions. These galvanostatic discharge values were used to determine the specific capacitance, energy, and power densities of the cells. Figure 10a,10d presents the corresponding capacity enhancements under the evaluated galvanostatic specific current rates. The specific capacities of the Ge–C_2_–CN photo-E ZIC at all examined specific current rates are higher than those of the Ge–H photo-E ZIC. This enhancement could be due to the presence of nitrogen and carbon in Ge–C_2_–CN which they typically incorporate into the electrode materials of an energy storage device to enhance their electrical and electrochemical properties. In addition to this, higher interlayer spacing of the Ge–C_2_–CN sample compared to that of Ge–H can provide adequate sites for the adsorption of ions from the ZnSO_4_ electrolyte, thereby enhancing the capacitance of the capacitor. The representation of the CD analyses of the photo-E ZICs in dark and illumination conditions at specific current rates of 50 and 60 mA g^–1^ are shown in Figure 10b,c,10e,10f. The results demonstrate that in the dark status, the charge and discharge times of the Ge–C_2_–CN photo-E ZIC are slightly higher than those of the Ge–H cell. However, the charge and discharge times of the Ge–H photo-E ZIC are greater than those of the Ge–C_2_–CN under illumination conditions. This behavior is due to the larger photogenerated electron and holes in the Ge–H cell which has already been confirmed by CV analyses.

**Figure 11:**
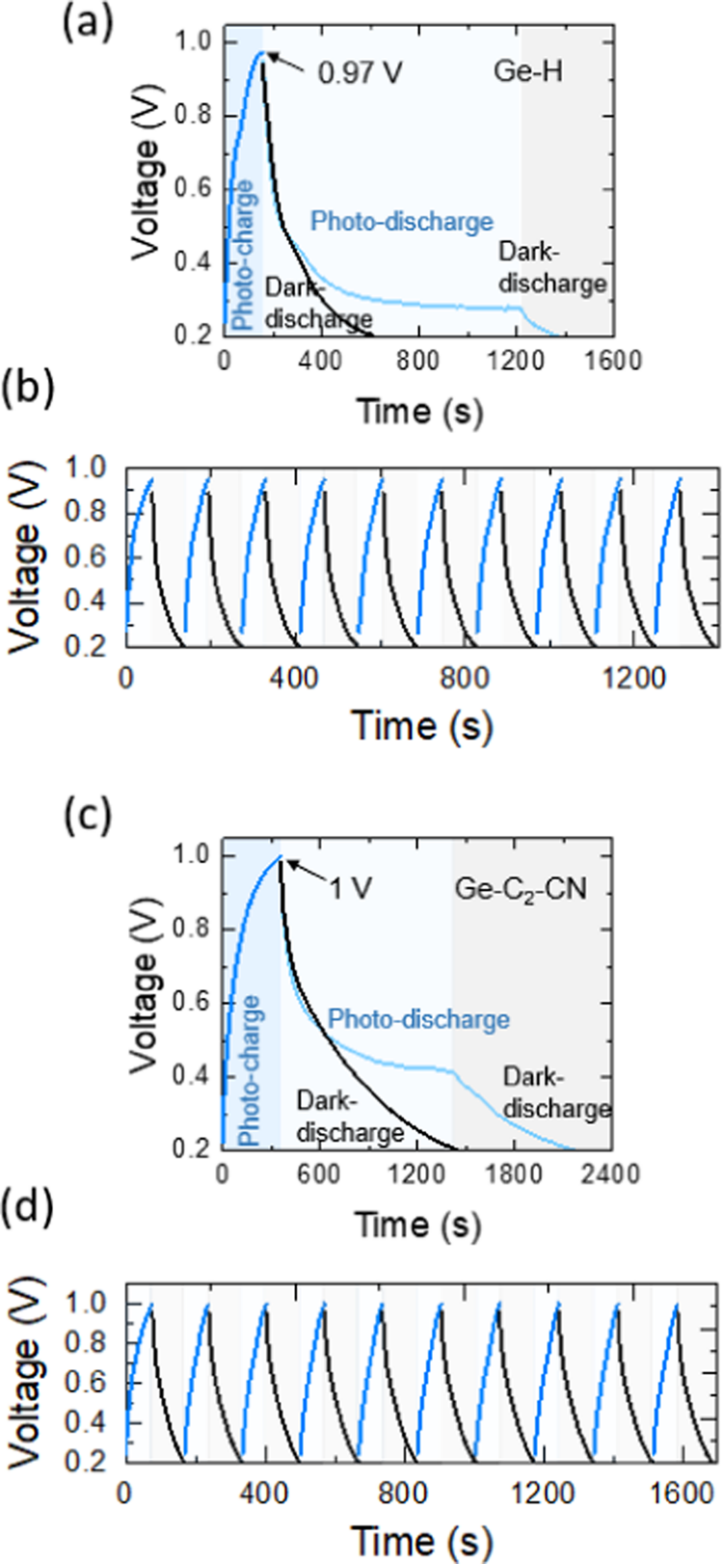
Photocharged and photodischarged (λ = 435 nm, 100 mW cm^–2^, at 10 mA g^–1^) and dark-discharged (at 10 mA g^–1^) behavior of (a) Ge–H and (c) Ge–C_2_–CN photo-E ZICs. Cyclic photocharged (λ = 435 nm, 100 mW cm^–2^, at 10 mA g^–1^) and dark-discharged (at 10 mA g^–1^) behavior of (b) Ge–H and (d) Ge–C_2_–CN photo-E ZICs at their initial 10 cycles.

In order to evaluate the photocharge, photodischarge, and dark-discharge behaviors as well as photocharging voltage responses of the cells in detail, both of the photo-E ZICs were charged under illumination first and then were discharged at two conditions of illumination and dark by the same charge scan rates (Figure 11a,c). The influence of the existence and absence of light on charge and discharge processes is demonstrated in Figure 11a,c. According to these results, the photo-E ZICs benefit from a continuous influx of photons during the photocharge (and photodischarge) with the presence of light which facilitates the rapid formation of electron–hole pairs. The light energy in the photocharge state assists in enhancing the efficiency of charge transfer in addition to the applied positive specific scan rate of 10 mA g^–1^, leading to a quicker charging period compared to the photodischarging process where a negative specific scan rate of 10 mA g^–1^ is applying but light energy still is continuing to generate electron–hole pairs. However, the photovoltaic effect remains inactive in the dark mode without any ongoing generation of electron–hole pairs. As a consequence, the discharge process of the capacitor relies entirely on the stored charge process. Figure 11a,c also demonstrates that the Ge–H photo-E ZIC is getting photocharged quickly (∼157 s) and reaches a maximum output voltage of ∼970 mV, while the Ge–C_2_–CN photocharge time is larger (∼362 s) with a 1000 mV output voltage. These photocharge time and output voltage enhancements of Ge–C_2_–CN photo-E ZIC are probably because of the larger interlayer spacing in this specimen with the benefit of accommodating greater charge carriers. The photodischarge time trend is in line with photocharge time when the Ge–H photo-E ZIC reaches ∼0.3 V after ∼1043 s; however, the Ge–C_2_–CN photo-E ZIC attains ∼1047 s before it reaches ∼0.4 V and getting stable. The galvanostatic dark-discharge times of the Ge–H and Ge–C_2_–CN photo-E ZICs are much quicker in both samples, and they attain the initial states of 0.2 V after ∼466 and ∼1068 s, respectively. In summary, these findings indicate that the photocharge and photodischarge activities of Ge–C_2_–CN are better than Ge–H in the context of storing energy capability. Moreover, the output voltage values of these photo-E ZICs are comparable to those of the most recent photo-E energy storage systems with a single architecture summarized in Table 1. Figure S8 shows the photocharging status of the Ge–H and Ge–C_2_–CN photo-E ZICs under a voltage-floating with zero applied current. They indicate the maximum charged values of the samples before getting constant during the voltage-floating tests. These results also confirm the higher charged value of the Ge–C_2_–CN photo-E ZIC (Figure S8b) at a shorter time compared to the Ge–H photo-E ZIC (Figure S8a), which is consistent with the former results. Initial 10-cycle stability was also assessed on the photo-E ZICs by subjecting them to repeated photocharging with λ = 435 nm and intensity of 100 mW cm^–2^, followed by galvanostatic discharging at a scan rate of 0.2 mA g^–1^ (Figure 11b,11d). A constant and regular photocharging movement was observed during the cycling on both photo-E ZICs.

**Table 1:** Comparing the Results of Some Previously Reported Cutting-Edge Photoenhanced Supercapacitors and Hybrid Capacitors with the Results of This Research Work

photoenhanced energy storage system		electrolyte	architecture	illumination	specific capacitance	energy and power densities	voltage response	photoenhancement efficiency	refs
supercapacitor	carbon dots modified Ti_3_C_2_T_*x*_-based fibrous supercapacitor	1 M H_2_SO_4_	single	400 nm ≤ λ ≤ 800 nm, intensity ∼150 mW cm^–2^	630 F g^–1^@10 A cm^–3^	18.75 mWh cm^–3^ and 8382 mW cm^–3^	∼400 mV	∼35.9%	(64)
	nickel–cobalt-deposited tungsten-doped TiO_2_ nanotube supercapacitor	3 M KOH	single	visible, intensity ∼100 mW cm^–2^	∼75.2 mF cm^–2^@0.04 mA cm^–2^	33.93 × 0^–3^ Wh cm^–2^ and 7.54 × 10^–3^ W cm^–2^@0.04 mA cm^–2^	∼450 mV	∼46%	(65)
	BiVO_4_–V_2_O_5_@titania nanotubes supercapacitor	3 M KCl	single	visible, intensity ∼100 mW cm^–2^	∼ 288 mF cm^–2^@0.12 mA cm^–2^	0.04 Wh cm^–2^ and 24 × 10^–5^ W cm^–2^@0.23 mA cm^–2^	∼150 mV		(66)
	Cu foam-supported CuO_*x*_@NiCuO_*x*_	2 M KOH	single	perfect light PLS-SXE300, intensity 1.76 W	2.64 F cm^–2^	∼8 Wh m^–2^ and ∼80 W m^–2^	∼600 mV	∼44%	(67)
	sulfur-, tungsten-doped TiO_2_ nanotube supercapacitor	0.5 M H_2_SO_4_	single	λ = 533 nm, intensity ∼100 mW cm^–2^	∼31 mF cm^–2^@0.23 mA cm^–2^	6.21 Wh cm^–2^ and 399 W cm^–2^@0.23 mA cm^–2^	∼400 mV		(68)
hybrid capacitor	zinc-ion capacitor using 2D g-C_3_N_4_	2 M ZnSO_4_	single	λ = 420 nm, intensity ∼50 mW cm^–2^	∼11,377 mF g^–1^@5 mA g^–1^	∼668 mWh kg^–1^ and ∼1625 mW kg^–1^@5 mA g^–1^	∼850 mV	∼82%	(63)
	ITO/PET (supercapacitor)	1 M LiPF_6_ salt in ethylene carbonate and diethyl carbonate (1:1; v/v)	single	intensity 100 mW cm^–2^	127 mAh g^–1^ (after 1000 cycles) 1A g^–1^	∼40 Wh kg^–1^ and 2000 W kg^–1^	∼3 V	∼8.41%	(69)
	zinc-ion capacitor using Ag@V_2_O_5_-activated carbon	3 M Zn(CF_3_SO_3_)_2_	single	λ = 455 nm, intensity ∼12 mW cm^–2^	∼138 F g^–1^	∼53.13 Wh kg^–1^ and ∼36.74 W kg^–1^	∼500 mV	∼63%	(70)
	Mg-ion capacitor using VO_2_	1 M Mg(NO_3_)_2_	single	λ = 455 nm, intensity ∼12 mW cm^–2^	∼63.80 F g^–1^@0.65 A g^–1^	∼20.5 mAh kg^–1^	∼70 mV	∼56%	(71)
	zinc-ion capacitor using germanane	2 M ZnSO_4_	single	λ = 435 nm, intensity ∼100 mW cm^–2^	∼ 4000 mF g^–1^@5 mA g^–1^	∼360 mWh kg^–1^ and ∼2000 mW kg^–1^@5 mA g^–1^	∼970 mV	∼52%	this work
	zinc-ion capacitor using cyanoethyl-germanane	2 M ZnSO_4_	single	λ = 435 nm, intensity ∼100 mW cm^–2^	∼6167 mF g^–1^@5 mA g^–1^	∼548 mWh kg^–1^ and ∼3091 mW kg^–1^@5 mA g^–1^	∼1000 mV	∼26%	this work

**Figure 12:**
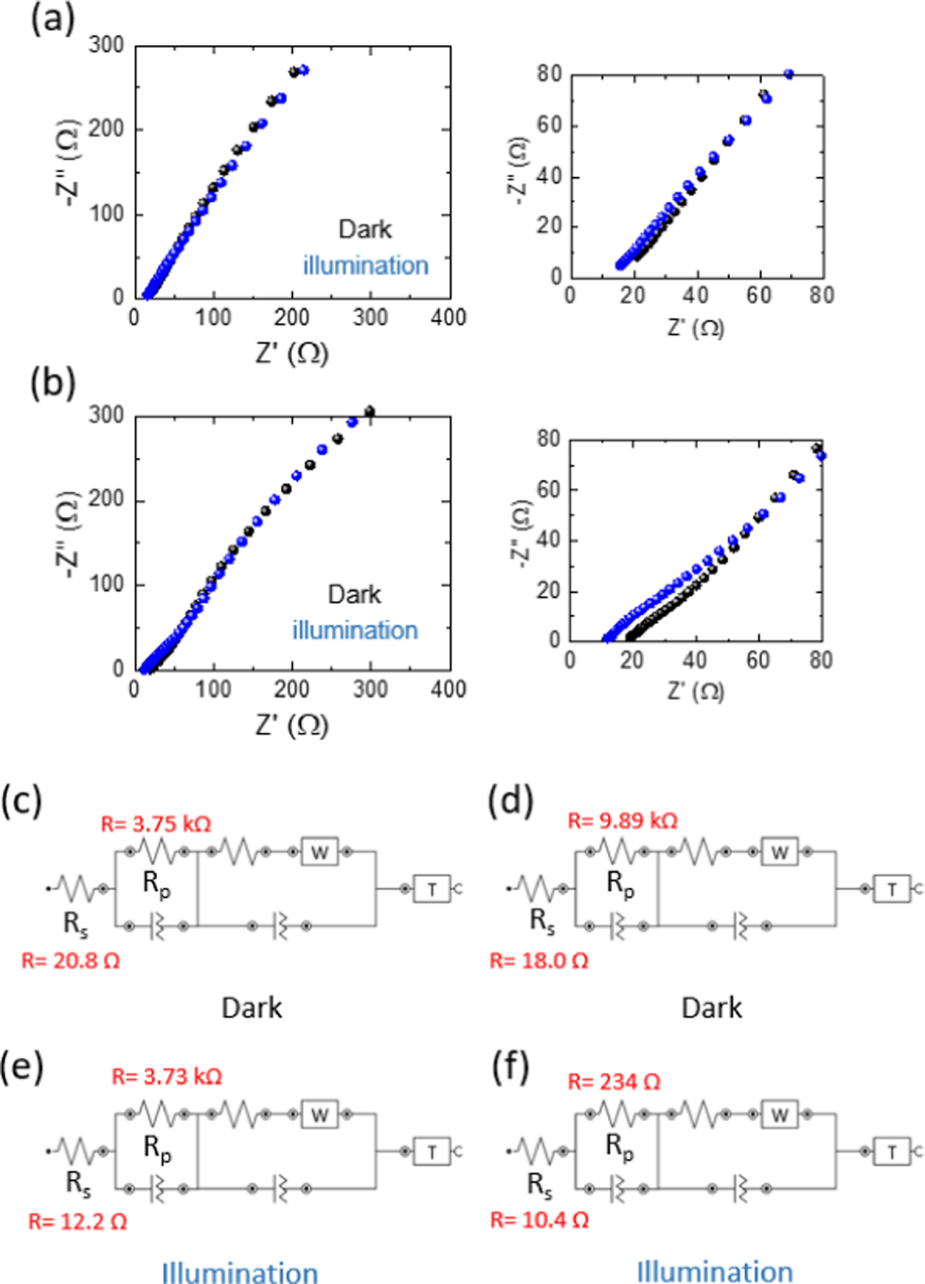
Nyquist plots and their equivalent circuit diagrams of (a, c, e) Ge–H and (b, d, f) Ge–C_2_–CN photo-E ZICs in dark and illumination (λ = 435 nm, 100 mW cm^–2^, 0 V) conditions. Note: errors (χ^2^) of the fitted equivalent circuit diagrams are ∼0.5 for Ge–H dark, ∼0.06 for Ge–H illumination, ∼0.09 for Ge–C_2_–CN dark, and ∼0.08 for Ge–C_2_–CN illumination.

To investigate the charge transport and ion diffusion characteristics, the Nyquist impedance spectra of the Ge–H and Ge–C_2_–CN photo-E ZICs were analyzed as well. Figure 12a,b reveals that when the cells are exposed to light, photocathodes exhibit a lower charge transfer resistance. This phenomenon is evident from the shift of overall resistance toward lower values under illumination (*R*_s_ = ∼12.2 Ω for Ge–H and ∼10.4 Ω for Ge–C_2_–CN) compared to the dark situation (*R*_s_ = ∼20.8 Ω for Ge–H and 18.0 Ω for Ge–C_2_–CN). The Nyquist plots also were fitted, and their equivalent electrical circuit (Randles circuit) diagrams are illustrated in Figure 12c–f. According to the fitted results, all of the circuits begin with the active electrolyte resistance (*R*_s_) in series with a parallel combination of the constant phase element (CPE) and charge transfer resistance (*R*_p_), which are associated with the photocathodes. The second section of each sample has the previous elements, as well as, an impedance (*W*) representing a faradaic reaction and a tangent hyperbolic (*T*) element which are interconnected. These latter elements play a role in determining ion diffusion, transport, and kinetics processes within the electrolyte. It is noteworthy that the plots were well-fitted with low errors, specifically χ^2^ = 0.49 (dark) and 0.059 (illumination) for Ge–H and 0.094 (dark) and 0.083 (illumination) for Ge–C_2_–CN.

**Figure 13:**
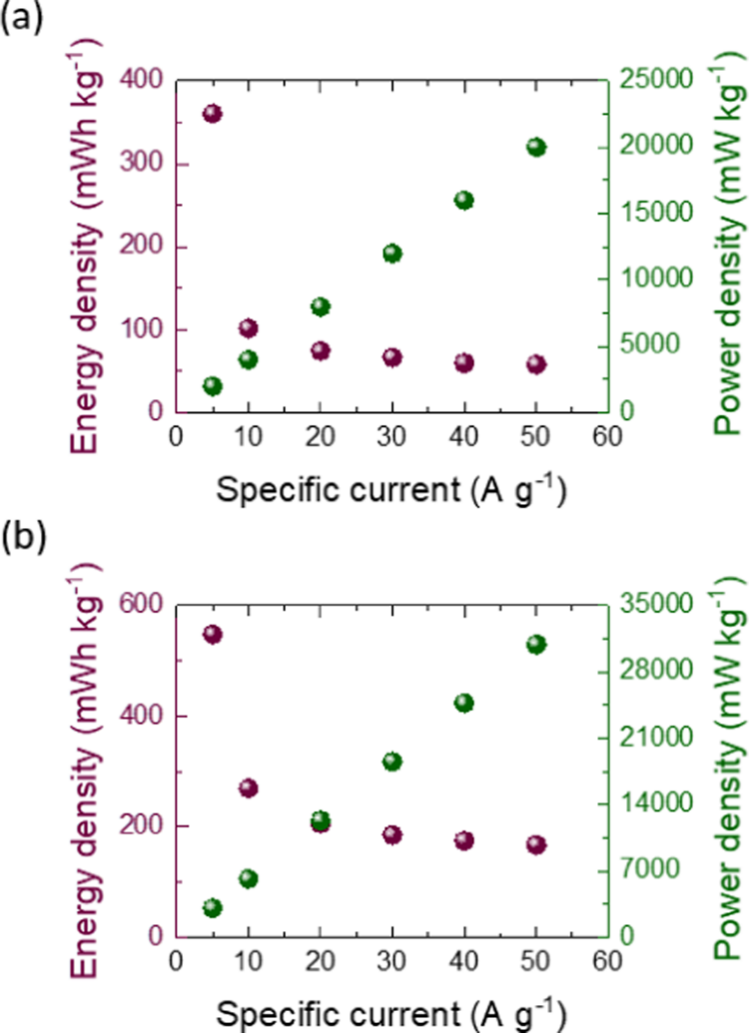
Calculated energy and power densities at different specific currents under dark conditions for (a) Ge–H and (b) Ge–C_2_–CN photo-E ZICs.

**Figure 14:**
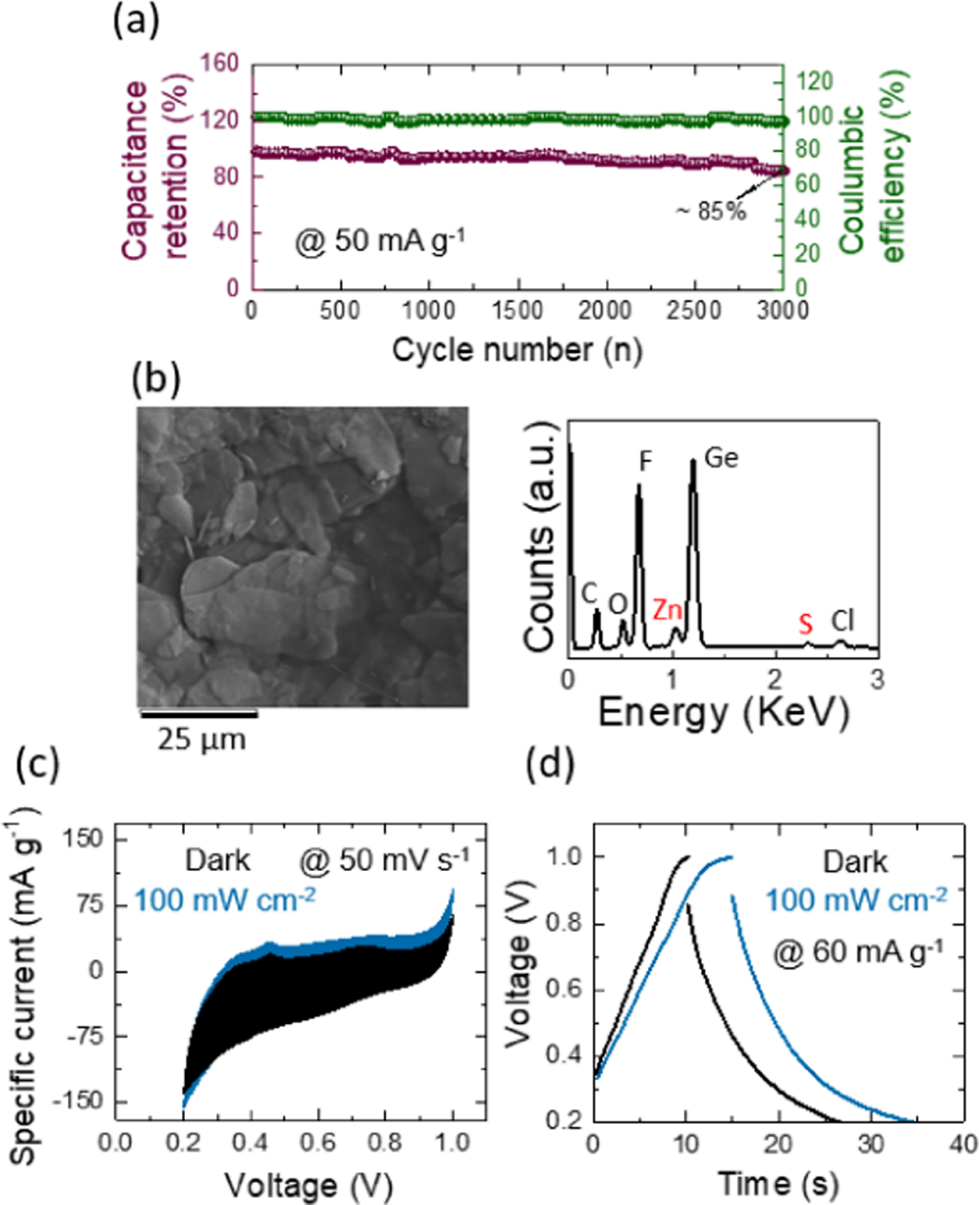
(a) Long-term cyclic stability of the Ge–H photo-E ZIC at a current density of 50 mA g^–1^ over 3,000 cycles. (b) SEM and EDS analyses, (c) CVs at 50 mV s^–1^ under dark and illumination conditions with 100 mW cm^–2^, and (d) CDs at 60 mA g^–1^ under dark and illumination conditions with 100 mW cm^–2^ for Ge–H photo-E ZIC after 3,000 life cycling stability.

**Figure 15:**
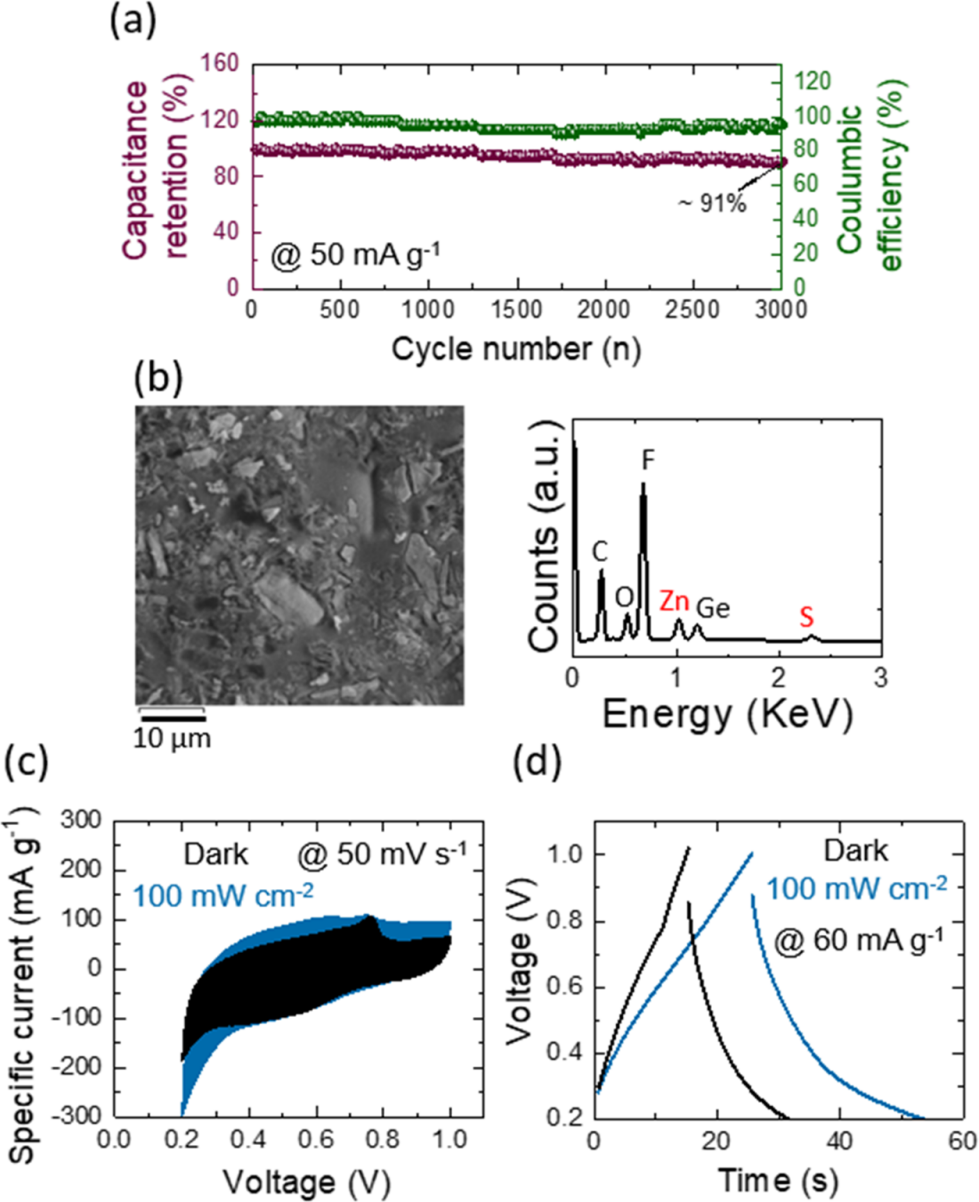
(a) Long-term cyclic stability of the Ge–C_2_–CN photo-E ZIC at a current density of 50 mA g^–1^ over 3000 cycles. (b) SEM and EDS analyses, (c) CVs at 50 mV s^–1^ under dark and illumination conditions with 100 mW cm^–2^, and (d) CDs at 60 mA g^–1^ under dark and illumination conditions with 100 mW cm^–2^ for Ge–C_2_–CN photo-E ZIC after 3000 life cycling stability.

The energy density and power density of the Ge–H and Ge–C_2_–CN photo-E ZICs were calculated in dark positions at different specific currents (Figure 13a,13b). It is seen that both energy and power densities have been developed in Ge–C_2_–CN photo-E ZIC. These enhancements are due to the existence of carbon and nitrogen elements in the Ge–C_2_–CN sample. Commonly, when carbon is introduced into the structure of supercapacitor electrodes, it can enhance the overall capacity of the device as this effect was already observed during the capacity assessment of this work as well. This is because carbon elements can provide additional sites and increase the surface area available for charge storage. As a result, the energy density, which is the value of energy that can be stored in a given volume or weight, can be enhanced. Carbon also can improve the power density of supercapacitor devices by developing their rate capability. The high electrical conductivity of carbon assists in having faster charge and discharge rates. This effect was also observed in photo-E ZIC made by Ge–C_2_–CN in comparison with the Ge–H device. Nitrogen also could develop the energy density of supercapacitor devices by modifying the electronic properties of electrode materials, making them more conducive, or altering their redox reactions. This leads to an enhancement in the capacity of the supercapacitor device, which, in turn, can increase the energy density. The power density of a supercapacitor device is impacted by nitrogen, primarily by influencing the kinetics of the charge/discharge processes. Nitrogen may show developed charge transfer kinetics and diminished internal resistance, which can result in higher power density and enabling faster energy release when required. It is crucial to note that the precise influences of carbon and nitrogen depend on some aspects, such as the concentration of such elements, the type of material being functionalized, and the overall design of the energy storage device. Thus, a careful strategy and material selection often could provide the right balance in the performance of an energy storage device. The long-term capacity retention and Coulombic efficiency of each Ge–H (Figure 14a) and Ge–C_2_–CN (Figure 15a) photo-E ZIC were executed over 3,000 cycles at a specific current of 50 mA g^–1^ in the dark position. It is observed from the data that both samples provide almost stable Coulombic efficiencies. The capacitance retention of the Ge–H photo-E ZIC is ∼85% after 3000 cycles, while the Ge–C_2_–CN photo-E ZIC affords ∼91%. This capacitance retention enhancement is due to the existence of carbon and nitrogen in the Ge–C_2_–CN structure, the effects of which on energy storage devices were discussed earlier. Morphology and EDS characteristics of Ge–H and Ge–C_2_–CN photocathodes after stability tests are provided in Figures 14b and 15b, respectively. The EDS results demonstrate a trace of sulfur and zinc belonging to the residual electrolyte and a sharp peak of fluorine due to the Nafion. A set of CV at a scan rate of 50 mV s^–1^ (Figures 14c and 15c) and CD at a specific current of 60 mA g^–1^ (Figures 14d and 15d) were operated on both photo-E ZICs in dark and illumination (100 mW cm^–2^) conditions to inspect photoenhancement efficiency of the devices after their stability tests. Although the photoenhancement efficiency of both photo-E ZICs has been reduced after their stability characterizations, they still contribute efficiently in response to the light during charge and discharge procedures.

## 4 Conclusions

This research presents a ground-breaking study on the development of dual-functional germanane-based photo-E ZICs with a novel approach utilizing Ge–H and Ge–C_2_–CN as photoactive materials. These materials enable direct charging through the absorption of light, eliminating the need for external photovoltaic devices. The investigation involved subjecting two distinct light intensities of 50 and 100 mW cm^–2^ with λ = 435 nm to both photo-E ZICs and comparing their performance under illumination with dark conditions. The promising results exhibit capacitance enhancements of ∼52% for Ge–H and ∼26% for Ge–C_2_–CN under an intensity of 100 mW cm^–2^. Additionally, the research achieved maximum output voltages of roughly 970 and 1,000 mV for Ge–H and Ge–C_2_–CN, respectively, at this level of light intensity. Further analysis of the fabricated photo-E ZICs revealed impressive capacitance retention rates, approximately 85% for Ge–H and 91% for Ge–C_2_–CN after undergoing 3,000 charge–discharge cycles. Those higher voltage responses, greater specific capacitance, and capacitance retention enhancement could be attributed to the incorporation of carbon, nitrogen, and larger interlayer spacing of the Ge–C_2_–CN. This study underscores the potential of conjugation conductive elements with the pioneer semiconductor 2D materials via a suitable chemistry procedure to not only supply a powerful single-architecture platform for new compact off-grid energy storage devices but also be a perspective to expand additional new nanostructures for the next-generation ultrathin and flexible photoenhanced energy storage devices. Moreover, the appropriate functionalization of conductive and semiconductor materials can strike the right balance in the efficiency of such unconventional photoenhanced energy storage systems, outperforming traditional integrated energy harvesting and storage approaches. The findings of this research open exciting avenues for advancing photodriven energy storage technology and promoting sustainable energy solutions.

## Data Availability

The datasets generated during and/or analyzed during the study are accessible via the Zenodo repository: 10.5281/zenodo.10814801.

## Supporting Information Available

Additional characterizations of the Ge–H and Ge–C_2_–CN materials including FTIR spectra; XPS spectra; SEM and EDS; SEM and EDS of their slurries containing GO; Raman spectra of GO; XRD parents of the slurries; CV loop for optimization; extra CVs; and voltage response of the photoactive cathodes (PDF)
