# Supplementary material for: Tuning Germanane Band Gaps via Cyanoethyl Functionalization for Cutting-Edge Photoactive Cathodes: Photoenhanced Hybrid Zinc-Ion Capacitor Evaluation
Source: ACS Appl Mater Interfaces. 2024 Mar 18;16(12):14722–41. doi: 10.1021/acsami.3c17420 (PMC10982940; doi:10.1021/acsami.3c17420)
Supplement: Supplementary file 1 — am3c17420_si_001.pdf [file am3c17420_si_001.pdf]

Supporting Information

Tuning Germanane Band Gaps via Cyanoethyl Functionalization for Cutting-Edge Photoactive Cathodes: Photoenhanced Hybrid Zinc-Ion Capacitor Evaluation

Jalal Azadmanjiri*, Jiri Sturala, Jakub Regner, Filipa M. Oliveira, Vlastimil Mazánek, Zdeněk Sofer*

Department of Inorganic Chemistry, University of Chemistry and Technology Prague, Technická 5, 166 28 Prague 6, Czech Republic

*Corresponding Authors: jalal_azad2000@yahoo.com, jalal.azadmanjiri@vscht.cz (Jalal Azadmanjiri); zdenek.sofer@vscht.cz (Zdeněk Sofer)

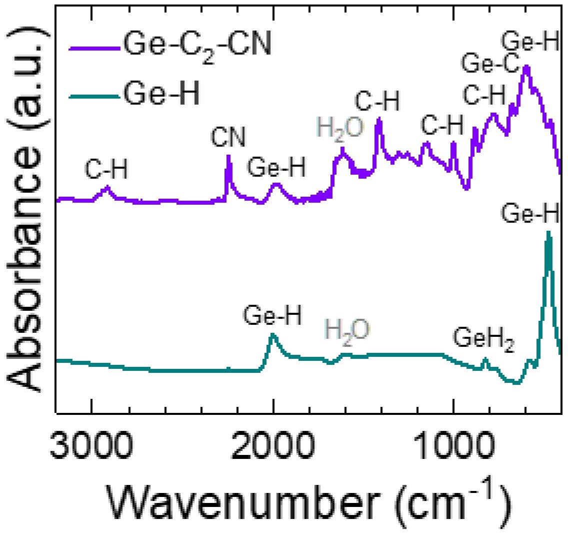

Figure S1. FTIR analyses of the Ge-H and Ge-C_2-CN powders.

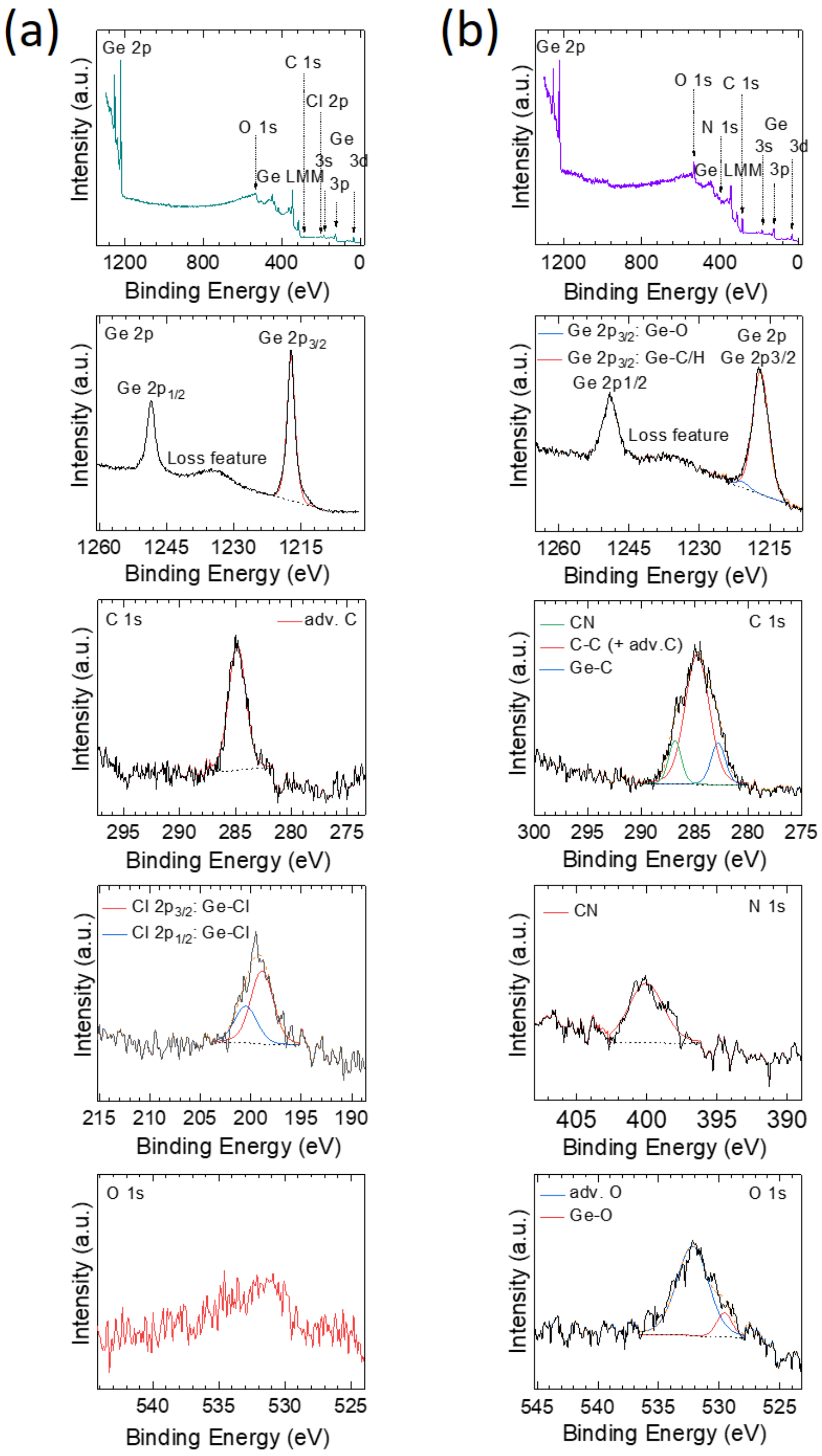

Figure S2. XPS survey and high-resolution spectrum analyses of the (a) Ge-H and (b) Ge-C_2-CN powders.

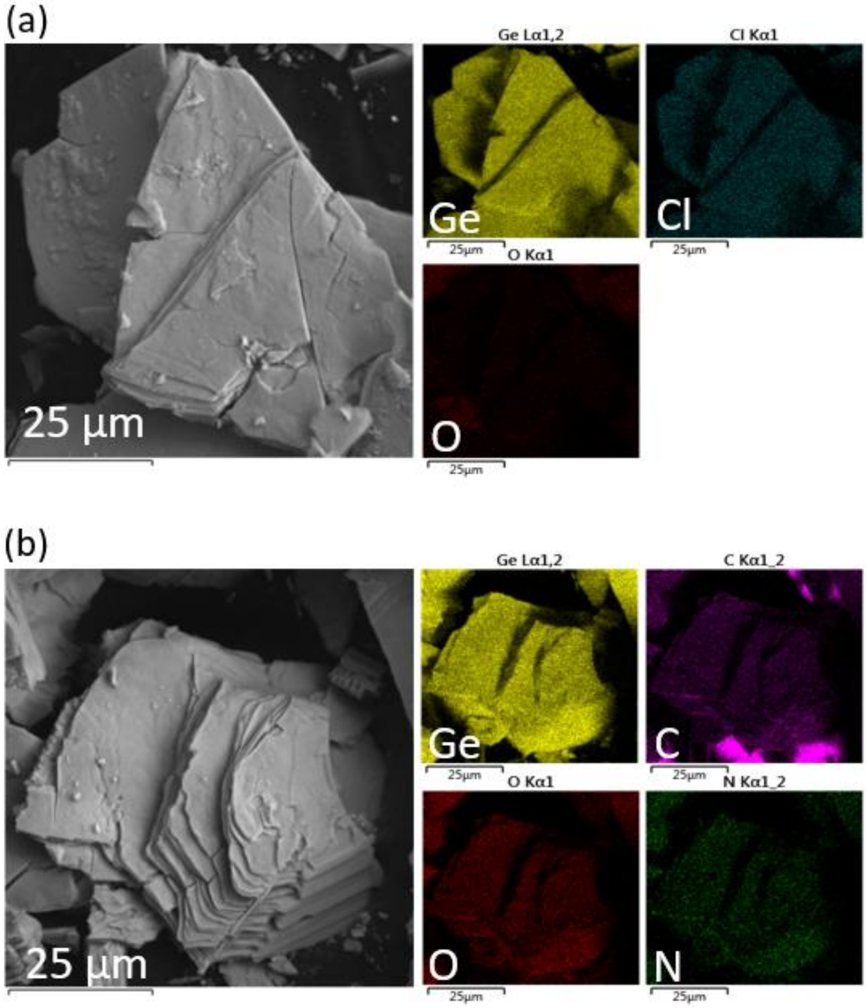

Figure S3. SEM and EDS images of the synthesized (a) Ge-H and (b) Ge-C_2-CN powders.

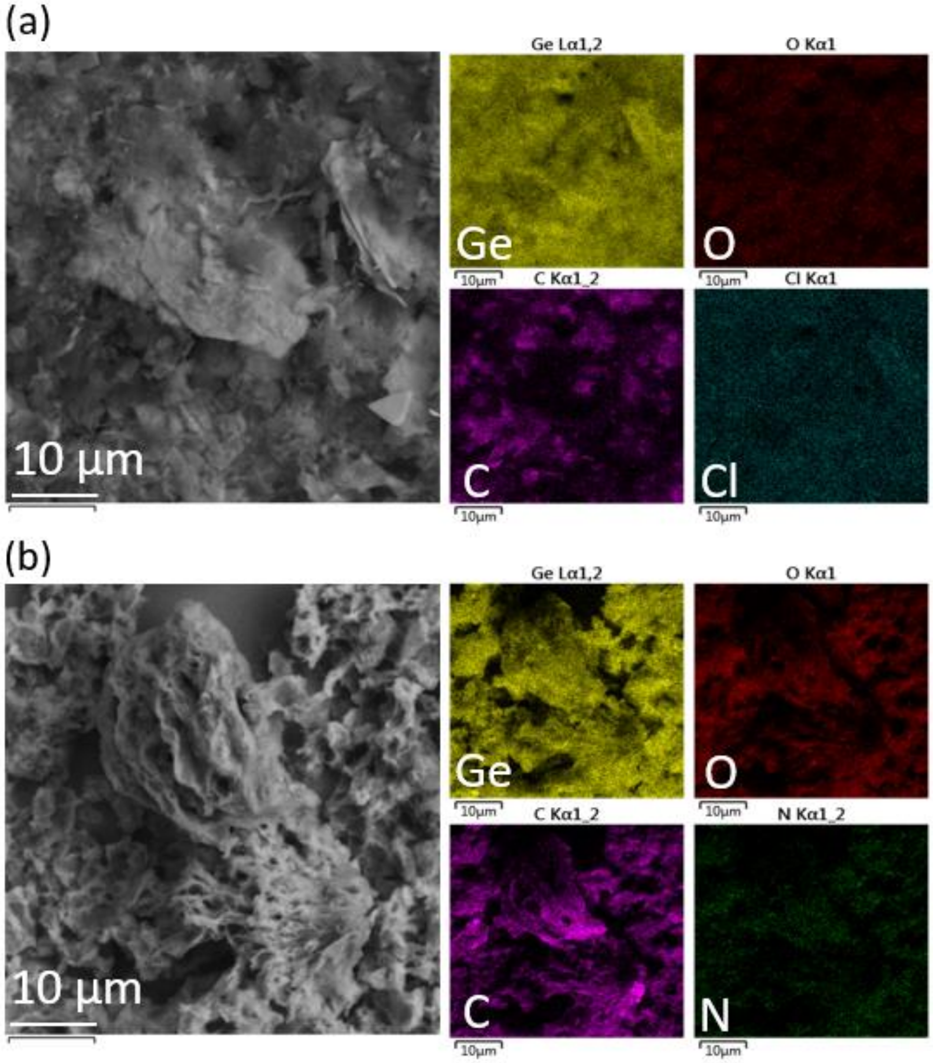

Figure S4. SEM and EDS images of (a) Ge-H and Ge-C_2-CN slurries contain of the additive GO.

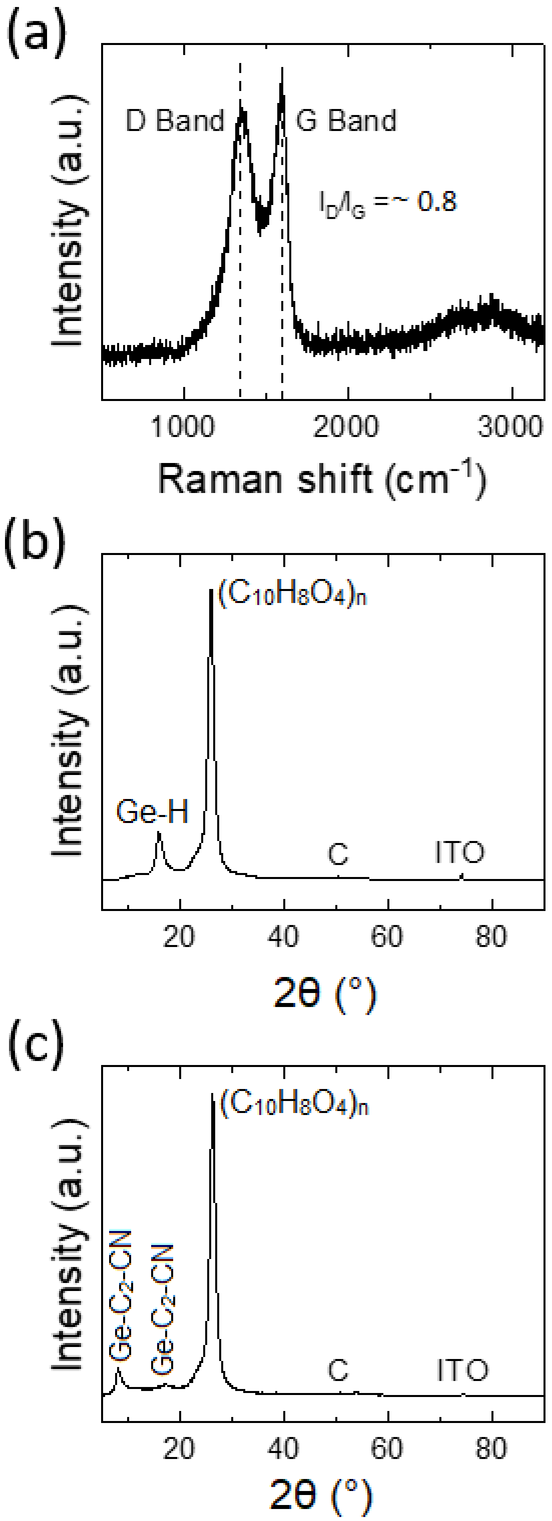

Figure S5. (a) Raman spectra of the additive GO, and XRD patterns of (b) Ge-H and (c) Ge-C_2-CN photocathodes.

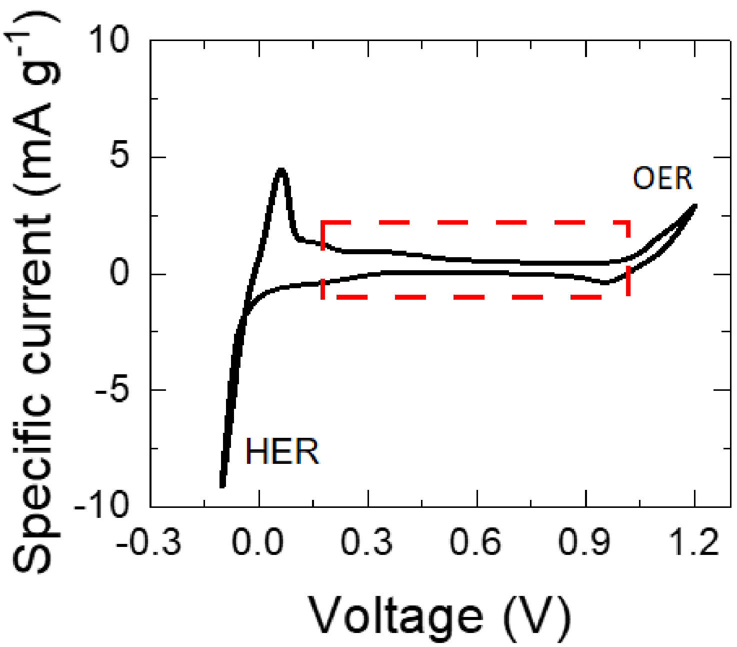

Figure S6. Examined CV on Ge-H photo-E ZIC at the voltage range -0.1 to 1.2 V (black) and a scan rate of 50 mV s^{-1} for optimizing CV range (red area) of photo-E ZICs during this study.

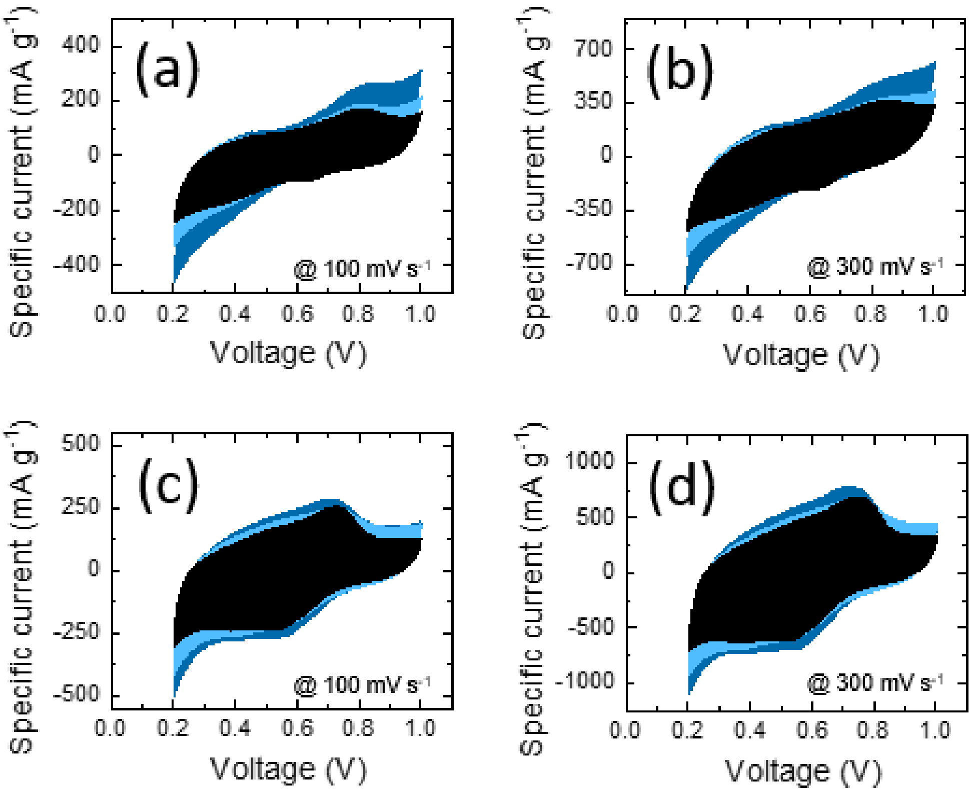

Figure S7. Comparative CV curves at different scan rates of 100, and 300 mV s^{-1} in dark, 50 mW cm^{-2} (light blue), and 100 mW cm^{-2} (dark blue) illumination with λ= 435 nm for (a and b) Ge-H and (c and d) Ge-C_2-CN photo-E ZICs.

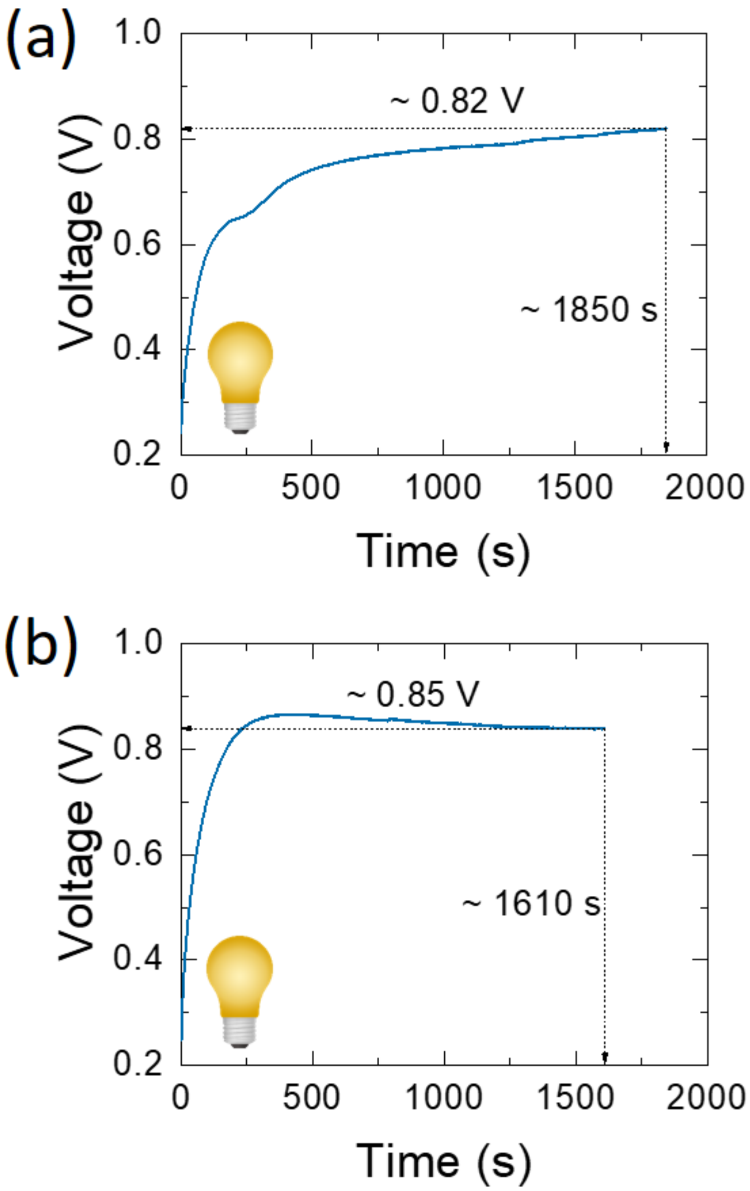

Figure S8. Voltage floating tests under continuous light (λ= 435 nm, 100 mW cm^{-2}) illumination at 0 A g^{-1} on (a) Ge-H and (b) Ge-C_2-CN photo-E ZICs.
